# SEAMM: A Simulation Environment for Atomistic and Molecular Modeling

**DOI:** 10.1021/acs.jpca.5c03164

**Published:** 2025-07-18

**Authors:** Paul Saxe, Jessica Nash, Mohammad Mostafanejad, Eliseo Marin-Rimoldi, Hasnain Hafiz, Louis G. Hector, T. Daniel Crawford

**Affiliations:** † Department of Chemistry, 1757 Virginia Tech, Blacksburg, Virginia 24061, United States; ‡ Molecular Sciences Software Institute, Blacksburg, Virginia 24060, United States; § Department of Chemical and Biomolecular Engineering, 101536 University of Notre Dame, Notre Dame, Indiana 46556, United States; ∥ Battery Research and Development, 6111 General Motors, Warren, Michigan 48092, United States

## Abstract

The simulation environment for atomistic and molecular modeling (SEAMM) is an open-source software package written in Python that provides a graphical interface for setting up, executing, and analyzing molecular and materials simulations. The graphical interface reduces the entry barrier for the use of new simulation tools, facilitating the interoperability of a wide range of simulation tools available to solve complex scientific and engineering problems in computational molecular science. Workflows are represented graphically by user-friendly flowcharts, which are shareable and reproducible. When a flowchart is executed within the SEAMM environment, all results, as well as metadata describing the workflow and codes used, are saved in a datastore that can be viewed using a browser-based dashboard, which allows collaborators to view the results and use the flowcharts to extend the results. SEAMM is a powerful productivity and collaboration tool that enables interoperability between simulation codes and ensures reproducibility and transparency in scientific research. We illustrate the flexibility and productivity of SEAMM with three examples: a simple molecular dynamics calculation to provide an overview; exploring the rearrangement of methylisocyanide to acetonitrile using a wide range of quantum codes and force fields; and using SEAMM for industrial research on battery materials with simulations of the diffusivity and ionic conductivity of electrolytes and the density, thermal expansion, and thermal conductivity of cathode materials as a function of lithiation.

## Graphical abstract

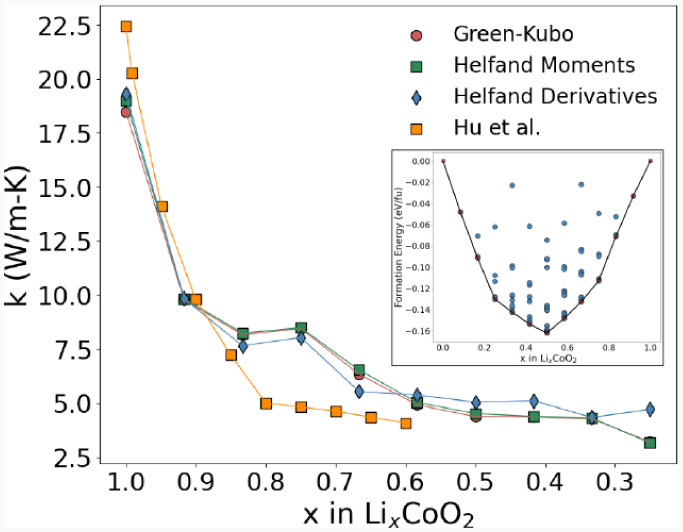

## 1 Introduction

Computational molecular science (CMS) and computational materials science (also CMS—we will use the term to encompass both) have been growing in importance since their beginning over 70 years ago. Figure 1 shows the growth of citations of the major CMS software packages in the international scientific literature as well as international patents, which are proxies for basic and applied research, respectively.

**Figure 1:**
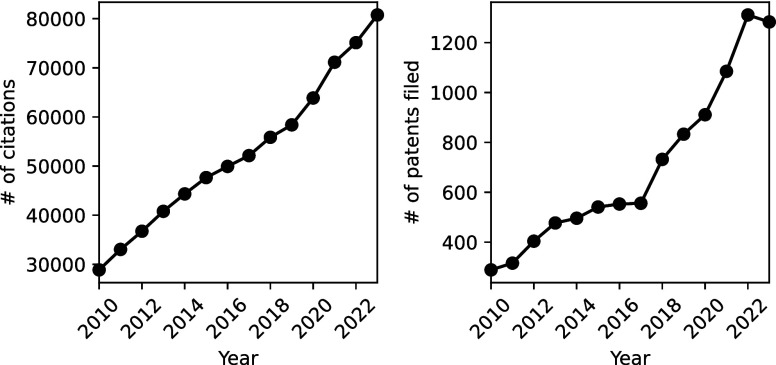
Number of citations^1,2^ (left panel) and patents (right panel) filed using significant CMS codes.

Citation statistics for the CMS codes, obtained from the atomistic.software website,^1,2^ are based on the Google Scholar tracking engine. The number of patents is also drawn from Google Patents. Similar trends are reported by other research groups,^3,4^ adopting bibliometric analysis of subjects such as density functional theory (DFT), which is the theory underlying a significant part of the CMS simulations.

The growth of annual citations of CMS codes is exponential with an approximate doubling time of about 9.5 years. The number of patents citing CMS codes is much smaller (≈1,300 vs 80,000 in 2023), but the growth is faster (doubling almost every 6 years). This is faster than the total scientific publishing counts, which is growing at about 4−5% per year with a doubling time of about 15 years.^5^ Clearly, both the development and application of CMS codes are of significant and increasing importance.

The growth of CMS as a field is mainly driven by two major factors. The technological factor, related to hardware and the incredible increase in available computing power, which currently has a doubling time of approximately 20 months.^6^ The computational factor, on the other hand, focuses on the continual development of the underlying theory and practical implementations in software. Over time, these two factors combine to allow researchers to perform a larger number of calculations and use more accurate but expensive methods, enabling a wider range of practical use cases.

How are CMS codes used in practice? Individual codes are often adopted to simulate a physical system, e.g., a molecule or collection of molecules, a fluid, a crystalline or amorphous solid, to predict its structure and properties, such as energy, band gap, or spectra. These simulations have many use cases. They can be used to explore systems of interest, to gain a better understanding, to help interpret and guide experiments, or to predict various properties that often inform downstream models. In some cases, a single code and type of simulation is sufficient to obtain a preset threshold of accuracy for simple and small systems. However, often numerous simulations are required, performed using different codes, to study and analyze the properties of larger and more complex systems. For example, optimizing the components of a battery might require quantum chemical simulations to determine reduction potentials, molecular dynamics simulations to predict diffusion constants and ionic conductivity of the electrolyte, and vibrational thermodynamics from phonon calculations to predict the entropy coefficient of anode and cathode materials for the reversible volumetric heat source term in battery thermal models.

It usually takes tens or hundreds of simulations to understand the system, discover the hidden patterns within a measured set of properties, and optimize these properties under certain conditions for practical purposes. Data management is also a significant challenge for simulations, as it requires careful tracking and documentation of the tools, methods, and parameters used. Furthermore, data management practices directly affect the reproducibility of the generated results and the extensibility of the simulations performed on new molecules or materials. At the end of the scholarly research process, downstream tasks such as writing reports, manuscripts, or patents summarizing the results of experiments will also be affected by data management practices.

Some projects are handled by a single researcher, but others may require a team of researchers with computational and experimental backgrounds in different parts of the problem. Some researchers might handle the simulations, while others use the results to interpret and assist experiments or as input to other models that operate at different length and time scales. This collaboration increases the need for careful organization and tracking of the artifacts of the simulations.

However, CMS codes are not easy to use: the underlying theory is complex, and the resulting equations are difficult to solve exactly except for the simplest ″toy″ systems, leading to many different approximations to the exact equations. Quantum chemistry codes have a range of methods from Hartree−Fock and DFT, which have larger approximations but are less computationally expensive, to coupled-cluster methods and full-CI, which are computationally very expensive but are closer to the exact solution. In DFT, the exact functional is not known, so there are numerous approximate functionals available. Most quantum codes make another approximation by expanding the wave function in terms of basis functions, which are typically Gaussian functions for molecules or plane waves for periodic systems. There are hundreds of different Gaussian basis sets in use.^7^ Molecular dynamics (MD) codes are also complicated with many choices of force fields or atomic potentials, many different algorithms to simulate different ensembles, and a wide variety of analyses. This complexity is unavoidable in any code trying to unravel chemistry and physics at the atomic level.^8^

This complexity is obvious in the input files that the user must prepare to run a simulation. There is a daunting number of input parameters to choose from. Some combinations of parameters may be allowed and others not, and if one parameter is set to a certain value, others may need to be adjusted to be compatible. Major quantum chemistry codes such as Gaussian^9,10^ and GAMESS^11^ have hundreds to a few thousand keywords to choose from. The Vienna Ab initio Simulation Package (VASP),^12–15^ an electronic structure code widely used in the materials field, has about 46 main keywords, many of which have suboptions, producing hundreds of combinations. This complexity is not restricted to quantum calculations: a large-scale atomic/molecular massively parallel simulator (LAMMPS),^16,17^ an MD code that is also widely used in the solid-state field, has over 1,000 commands, many of which have suboptions. It takes time and effort (and considerable trial and error) to learn how to use one of these simulation codes correctly and effectively. Unfortunately, while experience with one code reduces the barrier to learning a similar code, it only reduces it a moderate amount, because the input and output of different codes are quite different. Experience with one class of codes is very little help in learning to use a different type of code. Many experts in quantum chemistry codes are not comfortable with MD codes, and *vice versa*. Similarly, users of solid-state codes such as VASP face a considerable barrier with quantum chemistry codes and *vice versa*.

The same issues make it difficult to compare codes or reproduce or replicate the work of other researchers. It is challenging to include enough detail, even in the Supporting Information, to be able to reproduce the results using the same code. It is considerably more difficult to replicate results with a different code, since that requires good working knowledge of the original code to understand the details of the simulation and experience in the second code to set up a comparable simulation.^18^

These barriers impede all aspects of the CMS fields. The learning curve to become proficient with CMS limits the use of the tools and makes it more difficult to use multiple codes to address larger and more complex problems. Leveraging previous work to accelerate current work is less likely when the previous results cannot be reproduced. Both the learning curve and the difficulty with reproducibility make it more difficult for researchers to use the best tool for the problem at hand. It is hard to compare codes, and switching to a new code is costly, so there is a tendency to continue using known codes even if they are not as effective for a particular problem or if they are computationally more expensive. These barriers also slow the development of new and better codes for similar reasons.

Despite the issues, the use of CMS codes is increasing with time. Any steps that reduce the learning curve, let users access more of the wide range of powerful tools available, install the software more easily, and make results more reproducible and easier to compare between codes will benefit users and further accelerate the use of these codes on significant scientific and engineering problems. This will also benefit the community developing the codes, challenging them to strive for more capable and efficient software.

### 1.1 Existing Solutions

There are many efforts and codes that address the issues outlined above, indeed more than can be covered in detail here. This section will give examples of the most commonly used codes and how they fit the practical outline of how computational campaigns are structured. Starting at the lowest level, that is, the simulation codes themselves, progress is being made in standardizing parts of the input for quantum chemistry codes. For example, libxc^19^ is a library of DFT functionals currently used by more than 40 CMS software packages. libxc provides standard names for more than 600 functionals and ensures that the implementation is identical. Similarly, the basis set exchange (BSE)^7^ is an open library of Gaussian basis sets for quantum chemistry. The BSE defines a standard naming and implementation for more than 700 basis sets. libxc and the BSE are steps in the right direction but account for only two of the input parameters to quantum codes, although both parameters are key and have many possible values. The KIM project^20^ and the associated OpenKIM website^21^ are examples of efforts to standardize the atomistic potentials used by MD codes for materials science.

There are a number of codes for constructing and visualizing molecular and crystal structures. Some examples are JMol/JSMol,^22^ PyMol,^23^ RASMOL,^24^ VMD,^25^ OVITO,^26^ VESTA,^27^ and CrystalMaker.^28^ There is considerable variety in these codes. Some focus on molecular structures; others focus on crystal structures; and some on visualizing large-scale MD runs. Most are open-source, but CrystalMaker and PyMol are commercial products. Most, but not all, of PyMol is released under an open-source license. Some have tools for building structures, and a few can create the input files to run simulations with select codes. Building and visualizing structures is a key part of most simulations.

Another group of tools assists with setting up the input for calculations for a specific code or group of codes. Most can visualize systems, have building and editing tools for the structures, and provide a graphical user interface (GUI) for setting the parameters for the calculation. Avogadro^29,30^ is powerful and general, handling molecular and periodic systems, with good capabilities to build structures, analyze results, and interface with a number of codes through a plug-in architecture. Other graphical interfaces support a single code, such as GaussView^31^ for Gaussian, or for a set of similar codes, for example, Gabedit^32,33^ or Graphical Interface for Materials Simulations (GIMS).^34^ These codes are designed to handle one calculation at a time. A structure is read from a file or created using a builder, and then the calculation is set up and run. Many of the codes can read the output of the calculation and display the results graphically. For example, many of these tools for quantum codes can display orbitals and electron densities. The combination of graphical builders with GUIs to run calculations is an excellent approach to exploring and getting started on a new project. An advantage of setting up a calculation for a specific structure is that the interface can be tailored to the problem at hand, offering only relevant choices. However, using this type of code is tedious and error-prone for running calculations on more than a few structures, since the calculation must be manually set up and run for each new system. These codes handle more of the tasks needed for a computational campaign, but only cover some of the needed tasks.

A final class of codes, or environments composed of several codes, handle running many identical or similar calculations. Most of these codes provide a library of functions for common modeling tasks such as reading or building structures and running simulation codes. The user writes e.g., a Python script that sets the parameters and calls the library functions to run the calculations. This approach avoids the labor of repetitively setting up calculations for each structure and reduces the errors in manual approaches. Examples of these codes are AFLOW,^35^ AiiDA,^36,37^ automated quantum mechanical environments for researchers and educators (AQME),^38^ atomic simulation environment (ASE),^39^ atomate2,^40^ BigChem,^41^ Digichem,^42^ MoSDeF,^43^ pyiron,^44^ QCArchive,^45^ QMFlows,^46^ and wfl.^47^ Some of these codes, such as AiiDA and QCArchive store results in a database, organizing the results for easy access. Databases are a very powerful tool for organizing large data sets, but they are a challenge for most scientists to install and maintain, so database-centric environments are most useful for expert groups preparing large data sets for machine learning, force field development, or public databases.^48,49^ Environments that do not depend on a database, such as ASE, are easier for scientists to install and use, but they leave the organization of simulations and results to the user.

## 2 Methods

The general goal of simulation environment for atomistic and molecular modeling (SEAMM)^50,51^ is to facilitate and improve computational campaigns. In particular, SEAMM has the following goals:

1 Improve the productivity of users of atomistic simulation codes.
2 Make codes easier to use and less prone to errors.
3 Expand the range of codes that researchers can use by lowering the entry barrier.
4 Organize all inputs and outputs of simulations to aid users and collaborators in viewing and analyzing the results and to ensure reproducibility.
5 Track all workflows to ensure that results across a computational campaign are consistent and comparable and can be extended as needed.
6 Track all codes and auxiliary data, such as basis sets and force fields, used in the workflow, to help correctly cite and credit their authors.

In support of these goals, SEAMM can be used on a wide range of computers, from laptops to supercomputers that use Linux or MacOS operating systems. Although personal computers are both accessible and productive, they may not have the computational power for larger projects. However, SEAMM can be used across the network, so, for example, a user can setup simulations on their laptop, run the jobs on a remote supercomputer, and analyze the results back on their laptop.

### 2.1 Example 1

Before covering the components and architecture of SEAMM, a simple example will be illustrative from the user’s perspective. This example will show how to use MD in SEAMM to predict the density of ethanol at room temperature and pressure. At the heart of SEAMM are flowcharts, which encapsulate a reproducible workflow as a number of steps. Figure 2 shows a flowchart for creating a box of ethanol molecules as the model of the liquid and then using MD to predict its density.

**Figure 2:**
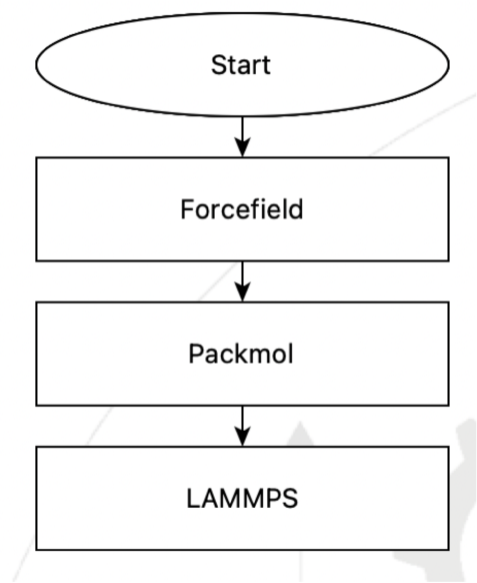
A sample flowchart for predicting the density of liquid ethanol via NPT MD simulation.

This simple flowchart has three steps: selecting the force field to use, building the liquid ethanol model using PACKMOL to create a cubic cell containing a few hundred molecules of ethanol, and then running a constant pressure MD simulation using LAMMPS.

The user interacts with two parts of SEAMM:

- A graphical editor that can retrieve flowcharts from libraries, create them from scratch, edit them, and submit them as a job to be executed.
- One or more web-based dashboards that handle the execution of the flowcharts and the data storage, tracking the jobs and monitoring of the results.

The flowchart in Figure 2 is created using the SEAMM GUI. The GUI provides a menu of steps. When the user adds a step, it is automatically added to the end of the flowchart queue, though it can be moved elsewhere if needed. The parameters for each step are edited using a dialogue window provided by the step.

The first step is the choice of force field. The dialogue for this step shows a list of available force fields, with the default being Optimized Potentials for Liquid Simulations − All Atom (OPLS-AA),^52^ which this example will use. For a force field such as OPLS-AA the correct force field atom types need to be assigned to the atoms in the structure. SEAMM automates this process by adding information to the force field file using SMARTS,^53^ which as its name suggests is closely related to SMILES,^54^ to describe the substructures associated with the various atom types. With this information, SEAMM fully automates the use of force fields without requiring any user intervention.

The dialogue window to configure the PACKMOL step parameters is shown in Figure 3.

**Figure 3:**
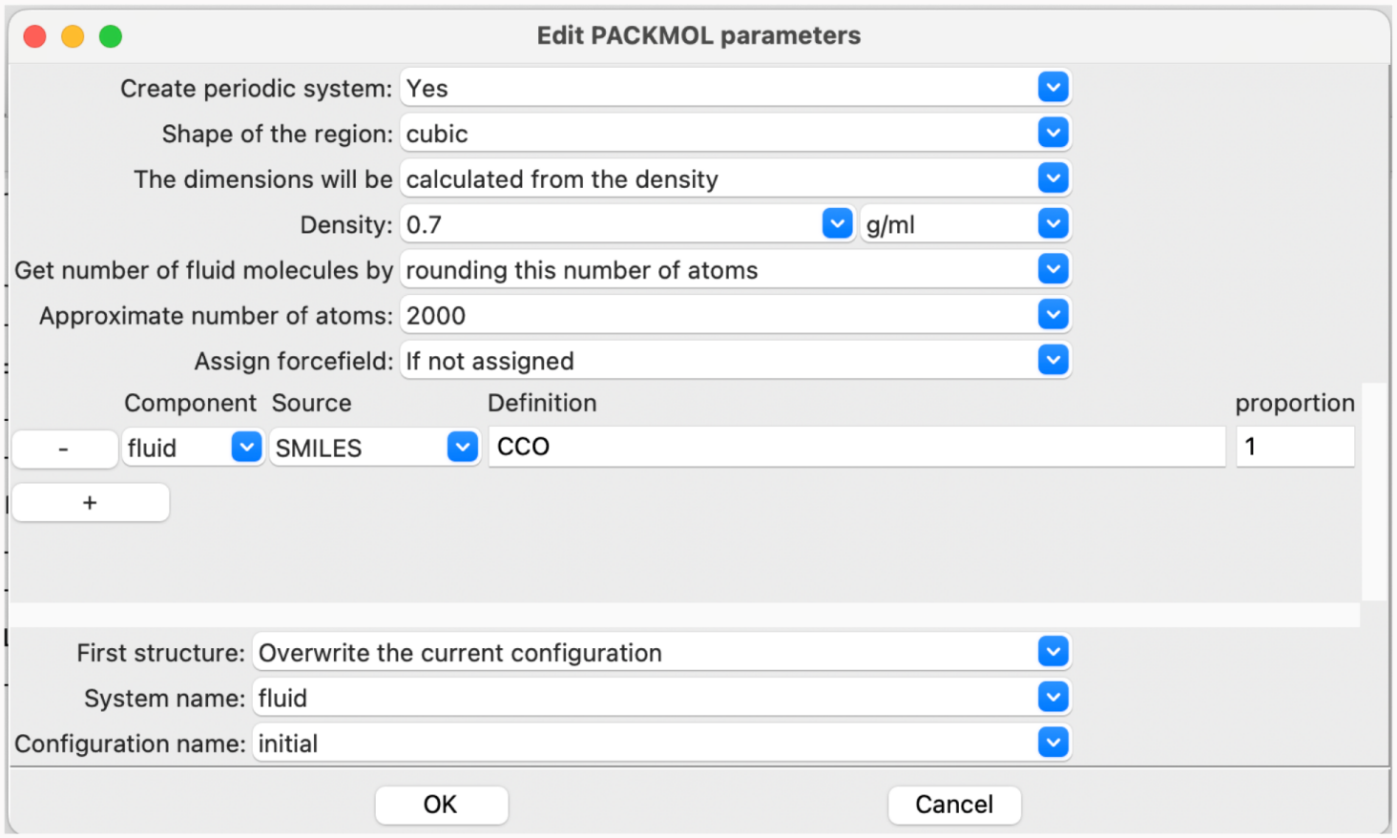
PACKMOL parameter configuration dialogue window in SEAMM.

In this example, the model is a cubic periodic cell containing a reasonable number of molecules of ethanol. The default values provided in the dialogue window are very important for usability. By default, the PACKMOL step builds a spherical droplet with the size calculated from the initial guess of the density specified in the dialogue and the number of molecules that contain about 2000 atoms. For this simulation, the first parameter in the dialogue window should be changed for a cubic periodic cell, but the default number of molecules is already set to a reasonable value for the selected cell type. It is well-known that some properties calculated for fluid simulations are sensitive to the cell size, converging with the size of the cell, though for many properties applying long-range corrections, which SEAMM does by default, mitigates this issue. However, the computational cost of MD simulations scales linearly with the size of the system, so the use of unnecessarily large cells increases the cost. The PACKMOL step uses this knowledge to guide and help users adopt good practices in their simulations. If the task was to compare the density of a number of compounds, say linear alcohols from methanol to decanol, SEAMM uses the number of atoms to determine the number of molecules, resulting in simulation cells of similar sizes. The simple approach of using the same number of molecules would result in the simulation for larger molecules using larger cells, which would make those simulations computational more expensive for no noticeable gain in precision. The design of the dialogue window and the default values in it guide users toward simulations that will provide reasonable, comparable results with minimum computational costs. The design also makes it straightforward to systematically change the size of the cell if there is any concern about the sensitivity of the property to the cell size.

**Figure 4:**
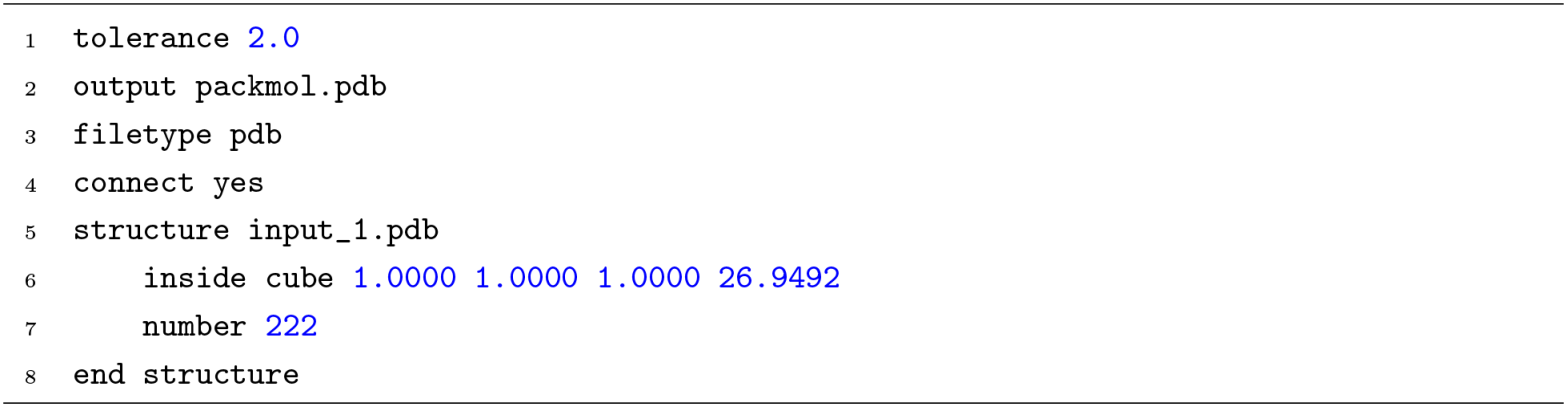
A sample input for PACKMOL.

Although the PACKMOL step in the SEAMM flowchart creates the input (Figure 4) to run PACKMOL for the user, its interpretation requires detailed knowledge of PACKMOL. Furthermore, the specific types of molecular structure files for input and output (here, PDB files) should be provided to PACKMOL by the user. In this example, SEAMM reads the SMILES^54^ of ethanol, CCO, generates the system model for subsequent steps. The PACKMOL step in SEAMM also prevents another issue. PACKMOL does not create periodic cells. The documentation for PACKMOL recommends creating a somewhat smaller cell of the desired shape and transforming the created structure into a periodic system of the correct size. Using a somewhat smaller structure avoids the creation of atoms that are too close to each other when the system is transformed into a periodic cell. For ethanol, the PACKMOL step in SEAMM specifies a cube of dimension 26.9492 Å, which is then transformed into a cubic periodic cell of dimension 28.9492 Å containing 222 ethanol molecules. The generated model system has an initial estimate for the density of 0.7 g mL^−1^ based on the user’s request.

Controlling the LAMMPS step is similarly straightforward. Since LAMMPS can handle multiple steps of a simulation in a single calculation, in SEAMM these internal steps are described by a subflowchart as shown in Figure 5.

**Figure 5:**
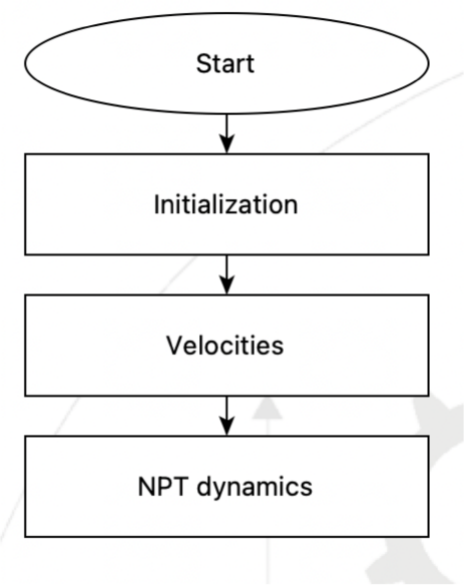
Subflowchart for LAMMPS.

The Initialization step establishes how to handle nonbonded interactions—van der Waals and Coulombic interactions—by providing a cutoff and, for periodic systems, whether to handle the long-range Coulombic terms with an Ewald summation, particle−particle particle−mesh (PPPM), or similar summation method, and also whether to correct for the long-range tail of the van der Waals terms. The default values of a cutoff of 10 Å and, for periodic systems, using the PPPM method and long-range tail correction are generally reasonable. The Velocities step prepares for the subsequent dynamics step by initializing the velocities on the atoms given a target temperature. The last step, the NPT step, controls the molecular dynamics as shown in Figure 6.

**Figure 6:**
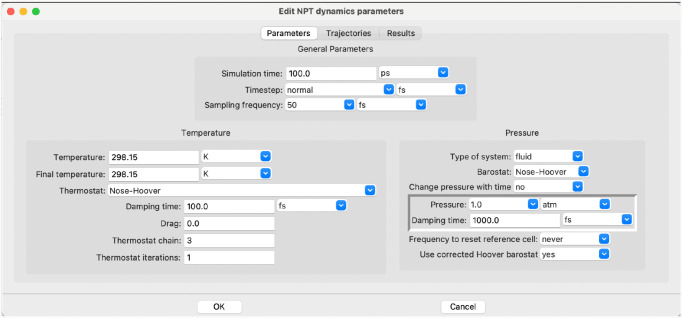
Parameters for LAMMPS.

Similarly to the PACKMOL step, the LAMMPS step also conceals considerable complexity and guides the user to a reasonable simulation. The input for LAMMPS is not shown here because the simulation control section in it is 115 lines of text and the data describing the molecular structure and the associated force field parameters add another 9415 lines. All input and output files from the job, including the LAMMPS step, are available in the Supporting Information.

Once the flowchart is ready, the user can submit it as a job to any accessible running SEAMM Dashboard. The Dashboard may be running on the local machine or a remote high-performance computing cluster intended for performing large-scale simulations. The job can be monitored on the Dashboard as it runs and the results examined as they become available. For analyzing the job results, SEAMM offers two useful view modes: a textual summary output, shown in Figure 7, and the diagrams of state variables and calculated properties, such as line plots of the density and autocorrelation function, shown in Figure 8.

**Figure 7:**
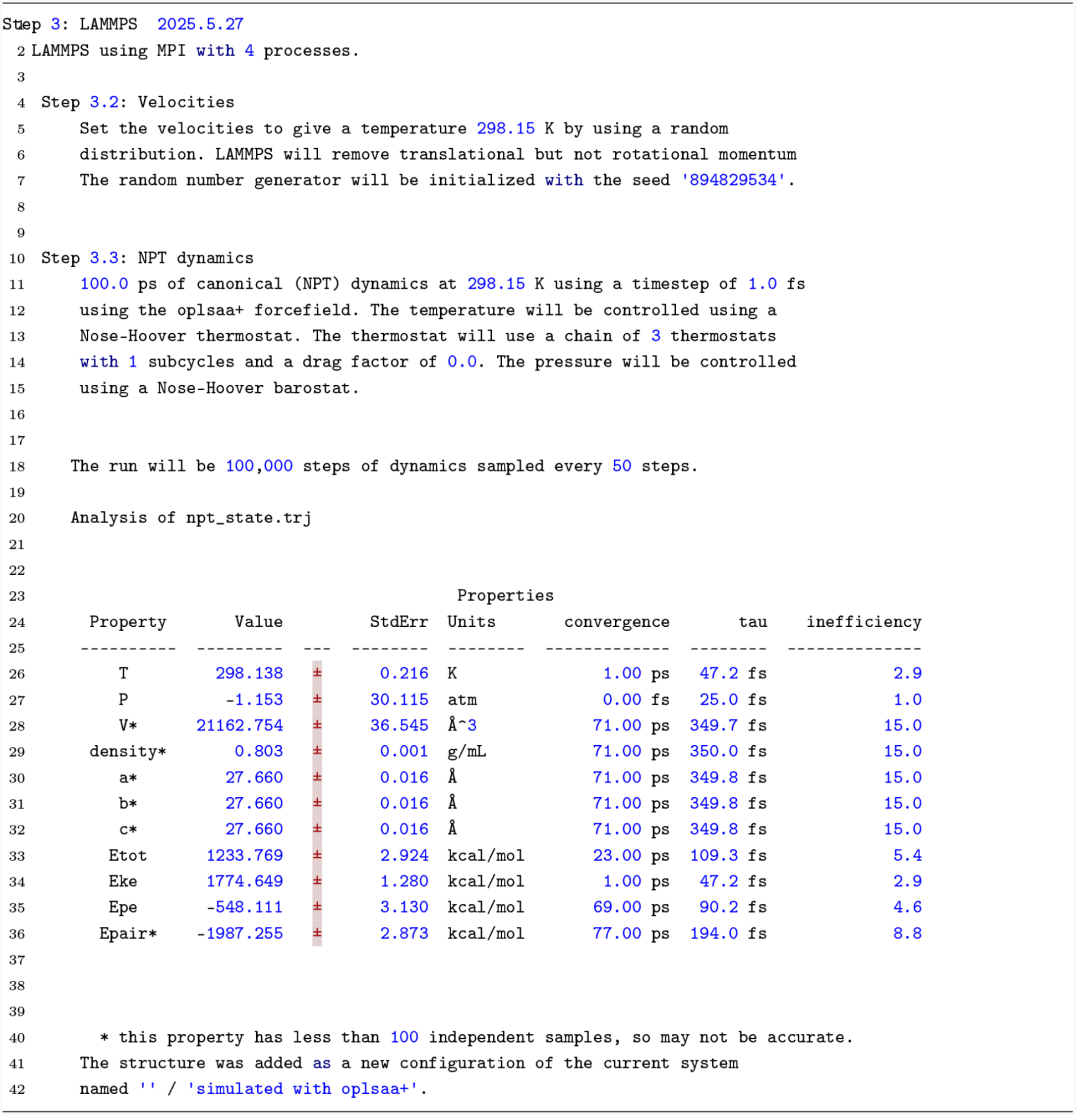
Summary output from the LAMMPS step.

The summary output view is available for any step in a flowchart. It starts with a brief description of the main operation performed within the step alongside the control parameters that define the simulation. Next, a short summary of the key results is presented. In the example above, a table of the properties, derived from the NPT simulation along with the statistical error bars, an estimate of when the property converged to a steady state, the time constant of the autocorrelation function (ACF), and statistical inefficiency of the sampling, are provided in the results section of the summary output. The calculated density value of 0.803 ± 0.001 kg L^−1^ at 298 K and 1 atm using the OPLS-AA force field compares well with the computed value 0.799 ± 0.002 in the original parametrization^56^ and is within about 1% of the experimental value at room temperature of 0.79.^57^

In addition to the summary outputs, the Dashboard often offers other files that can provide more graphical details on property analysis or structure visualization. In this example, SEAMM creates a diagram for each calculated property. The left panel in Figure 8 shows the line plot of the density simulation versus the time steps. Note the solid black line in the density diagram, indicating the average density calculated over the steady state simulation interval between 71 and 100 ps. The average density value is also presented in the textual output summary shown in Figure 7 above. The right panel in Figure 8 shows the ACF of the density in red with the error bounds shaded. The dark gray line is the best exponential fit to the ACF corresponding to the simulation time period reported in the summary output. These diagrams provide a rapid qualitative check on the calculation results presented in the summary output. Furthermore, the ACF and the accompanying statistical inefficiency metric, which is a measure of oversampling of the trajectory, provide key information to improve the simulation, if necessary.

**Figure 8:**
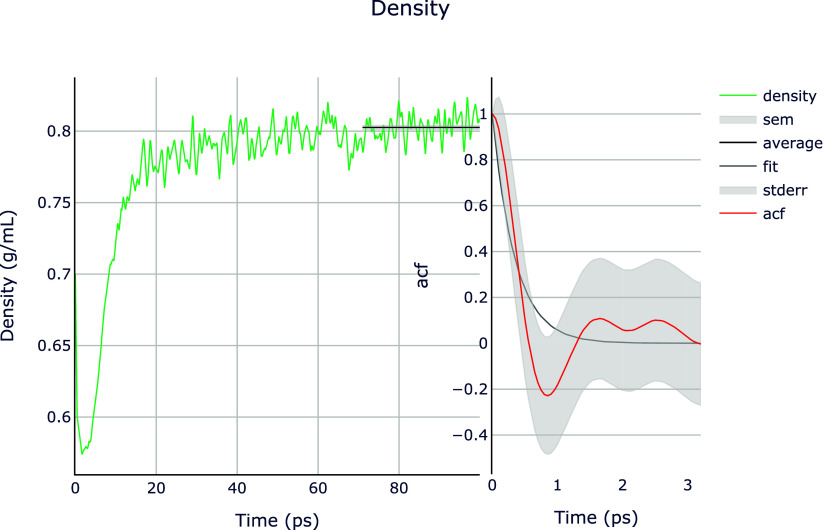
Line plots of the density (left) and the autocorrelation function (right) versus time, generated by the LAMMPS step in SEAMM. Note the horizontal line which SEAMM automatically creates on the density plot to represent the mean density value on the plateu.

Note that by changing the SMILES string in the PACKMOL step, the flowchart can be applied to other liquids, and mixtures can be simulated by adding additional molecules to the component table. SEAMM supports runtime variables in flowcharts, which are set on-the-fly when submitting the job and can be used anywhere in the flowchart instead of preset constants. Generalizing flowcharts using runtime variables enables SEAMM to predict the density of any liquid consisting of any molecules that the force field can handle.

SEAMM and the plug-ins provide the summaries of the steps in the workflow, as well as appropriate citations for the codes and parameter sets used, both as text and as BibT_E_X. This feature reduces the required effort for documenting the simulation details and providing a comprehensive description of the workflow. In the example shown above, the output and citations can easily be converted into the following sample description for scholarly publications: *The SEAMM environment* ^58–60^ *was used to predict the density of ethanol at 298.15 K and 1 atm via an MD simulation with an isothermal−isobaric (NPT) ensemble using a Nose-Hoover thermostat/barostat*,^61^ *running for 100 ps with a 1 fs time step using time-reversible, measure-preserving Verlet and rRESPA integrators* ^62^ *with the LAMMPS code.* ^16,63^ *The potential energy of the system was calculated using the OPLS-AA force field.* ^52^ *Nonbonded interactions were cut off at 10 Å with long-range Coulomb terms handled using a particle−particle particle−mesh solver* ^64^ *and a tail correction* ^65^ *for long-range Lennard-Jones terms. The model of liquid ethanol was a cubic periodic cell containing 222 molecules of ethanol. The initial structure was created using PACKMOL* ^66,67^ *to create a cubic cell with a density of 0.7 g/cm* ^*3*^ *using an ethanol structure created from SMILES using OpenBabel.* ^68,69^ *The analysis of the trajectory was carried out by the LAMMPS step* ^59^ *in SEAMM, using the pymbar library* ^70–73^ *to determine when the system had reached a steady state and to perform the statistical analysis of that portion of the trajectory.*

### 2.2 SEAMM Components

Figure 9 provides a high-level view of various parts of the SEAMM environment. The next subsections will provide more details on each component and how the individual parts work together to provide a comprehensive software ecosystem for CMS simulations.

**Figure 9:**
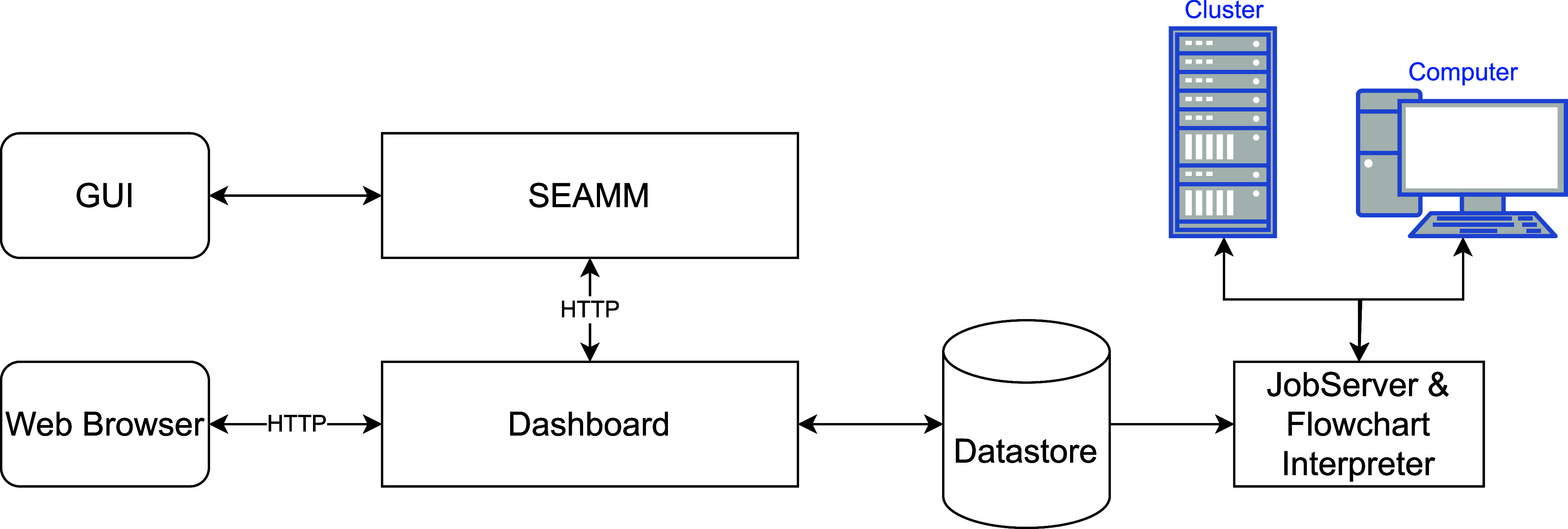
Components of the SEAMM environment.

#### 2.2.1 SEAMM Core

Figure 10 shows SEAMM, which is the central part of the SEAMM environment in Figure 9, in more detail. There are five parts: a database for storing computational results during a job, a GUI and utility libraries that contain useful functionality for developers, an application programming interface (API) that defines the interface between SEAMM and the plug-ins, which provide all the functionality that users see, e.g., the flowchart and dialogues in Example 1.

**Figure 10:**
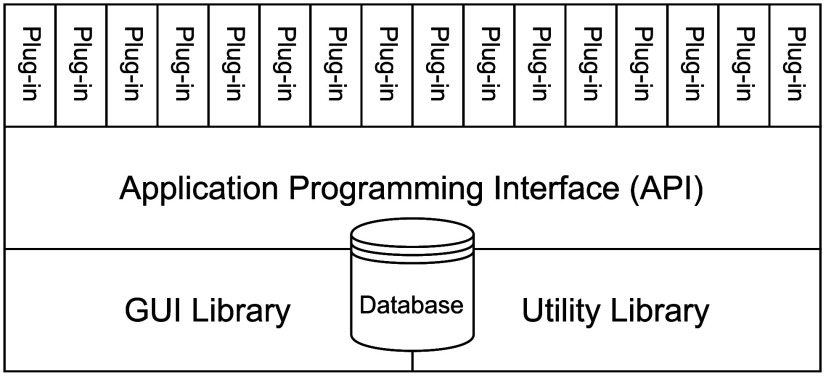
High-level software stack view of SEAMM core component.

#### 2.2.2 Plug-Ins

SEAMM, at its core, is a framework that provides its own data model and numerous utility functions without offering any end-user functionality. In order to enhance modularity, SEAMM directs external end-user functionalities into separate code units, called plug-ins, as shown in Figure 10. Each plug-in corresponds to a step in a flowchart. Plug-ins provide interfaces to external CMS simulation tools, builders for creating systems, analysis modules, control steps in flowcharts such as loops and subflowcharts, and other functionality that users need to run their simulations. Plug-ins are developed based on the SEAMM API but are independent software modules and distributed separately from SEAMM. The SEAMM installer finds the plug-ins, published on the Python Package Index (PyPI),^74^ and installs or updates them upon user request.

In Example 1, the three steps in the flowchart in Figure 2 correspond to individual plug-ins that provide interfaces for the PACKMOL, LAMMPS and the force field steps. When the flowchart is executed, the force field step plug-in reads the force field from a disk file and stores it in internal data structures for easy access. A substantial part of the utility library is devoted to handling force fields, including automatic “atom typing” and preparing the lists of force field parameters for codes such as LAMMPS. As such, the force field plug-in can automate most aspects of force field development for both users and developers, as detailed in Example 1, where the user only had to choose which force field they wanted to use, and everything else related to the force field was hidden.

There are currently 30 plug-ins available for the SEAMM environment. A list of the plug-ins along with a brief description of their functionality is included in the Supporting Information, along with a similar list of the 10 core components.

#### 2.2.3 Data Model and Internal Database

SEAMM supports simulations of molecules, fluids, and amorphous and crystalline solids plus interfaces between different phases. Although these structures are closely related, different disciplines use different terminologies which can be confusing. Before proceeding with discussing data storage, we will cover the adopted terminology in SEAMM. The term “*system*” denotes a collection of atoms, molecules, and/or crystals that are simulated, analogous to experiments on thermodynamic systems.^75^ In SEAMM, the *system* itself is an abstraction that consists of one or more *configurations*, which themselves are composed of atoms along with their coordinates, optional bonds between atoms, the periodic cell for periodic *systems*, the point- or space-group symmetries, and the charge and spin/magnetic states of the *system*. Many codes call what SEAMM refers to as a *configuration* a “molecule” or “structure”, or in some cases they are “conformers” or “frames” in a MD trajectory. The *configurations* in SEAMM are more general than the structures or the frames in trajectories in most codes. Different *configurations* of a *system* can have different atoms and different bonds. With this flexibility, SEAMM can support reactive dynamics and other simulations of chemical reactions in a natural way. SEAMM can also support grand canonical ensembles, where the number of atoms or molecules can change such as when simulating vapor deposition by creating a stream of new molecules to impinge on a target surface.

Most quantum codes take the charge and spin as input parameters for the simulation. In SEAMM, the charge and spin multiplicity are properties of the *configurations*. This feature extends the usability and generality of flowcharts to handle neutral molecules, anions, cations, and different spin states within the same framework. Chemists think of singlet and triplet oxygen as two different chemical entities, as does SEAMM.

SEAMM uses the concept of a *subset*, which is an arbitrary collection of atoms in a *configuration*. Atoms can be in more than one *subset*, which allows *subsets* to be defined for commonly used concepts such as residues and chains in proteins or adsorbents and adsorbates. *Templates* are closely related to *subsets* but contain a *configuration* that has atoms, bonds and coordinates, as well as other useful attributes such as names, partial charges, and force field atom types. A *template* is a prototype that can be used to find identical substructures in a *system*, matching them atom by atom regardless of the order of the atoms. Once matched, attributes such as atom names, partial charges, and bond orders can be transferred from the *template* to the substructure. For example, *templates* of the amino acids could be used to match the different residues in a protein and provide the structure with standard names, partial charges, and atom types.

The data for *systems*, *configurations*, *templates*, calculated properties and other results are stored in an internal relational database. Rather than accessing the database directly, the utility library provides a facade over the database, so for the rest of the SEAMM and to the plug-ins, the underlying data model is accessed through an object-oriented framework with *systems*, *configurations*, atoms, bonds, etc. This model is common in simulation codes, so developers will probably find this aspect of SEAMM similar to other software packages with which they may already be familiar.

There are several advantages to using a relational database. Relational Database Management Systems (RDBMSs) constitute a mature, well-understood, high-performance, and scalable technology. SEAMM currently uses SQLite^76^ for database management because it is small and lightweight, well integrated with Python, and designed for use within programs. Since structured query language (SQL) is a standard language for RDBMSs, including SQLite, SEAMM could switch to a different SQL-based RDBMS if needed. A second advantage, particularly of SQLite, is that the database file is portable across platforms, storing data with full precision fidelity. As such, SEAMM and the database files are also portable across different hardware and operating systems. Furthermore, the most important advantage gained is the flexibility and extensibility that a database provides.

The schema used in the database defines the data that it stores as tables, columns, and relations between them. There are two main parts of the schema used by SEAMM: a conventional schema for *systems* and *configurations*, and a star schema^77^ for property data and simulation results.

**Figure 11:**
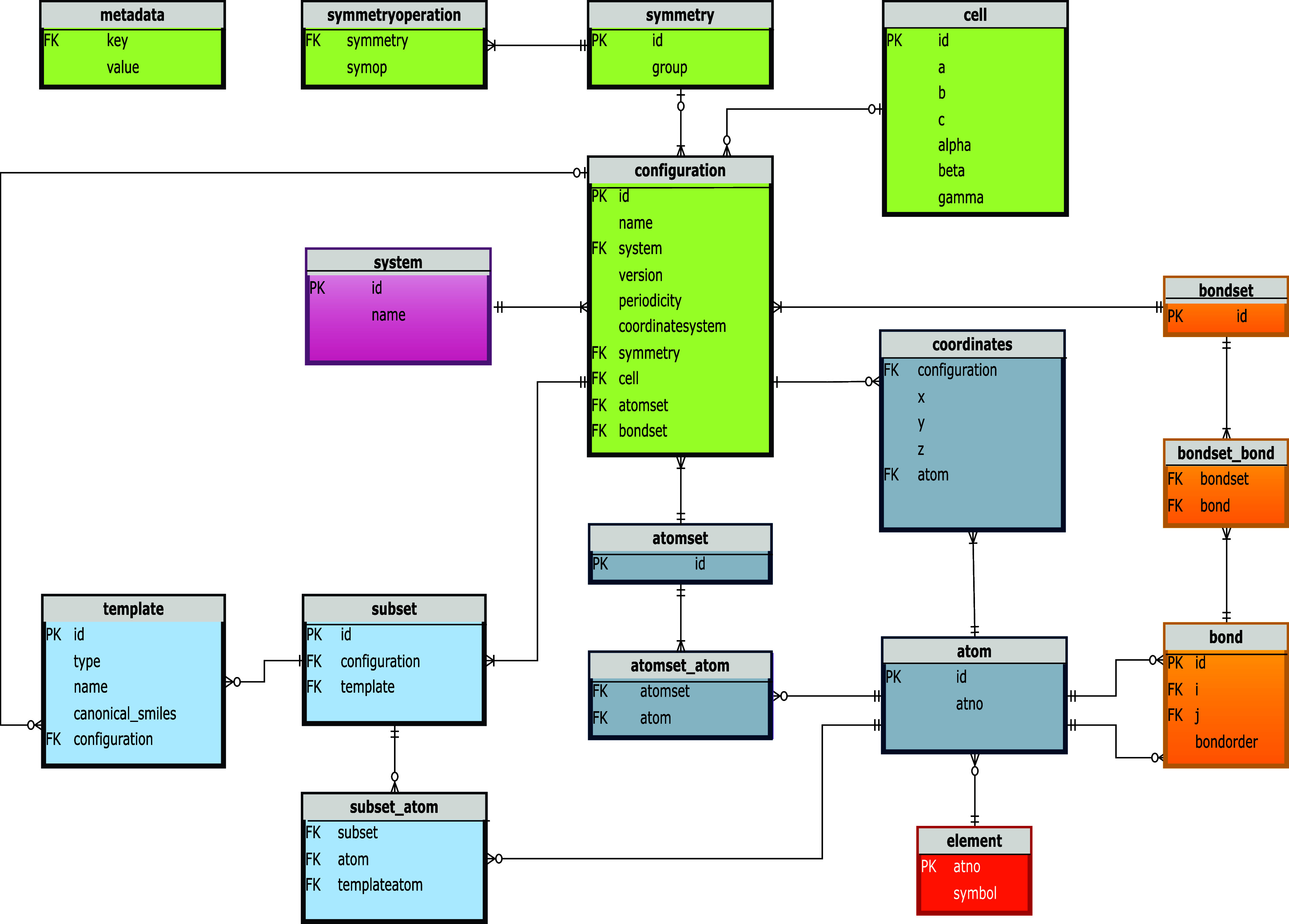
Database schema for molecular structures.

Figure 11 shows the schema for *systems* and *configurations*. The *configuration* is the heart of the description, connected to the atoms and bonds through the junction tables atomset_atom and bondset_bond. This allows *configurations* to contain different atoms or bonds, while avoiding the duplication of shared atoms and bonds to reduce the storage needed. The data storage size is important for large MD simulations with extensive trajectories. Storing the atomic coordinates separately from other atomic features also helps limit the amount of data stored during MD simulations, since in many simulations, the topology of the structure − atom types and bonds − do not change, but the atomic positions change with every step. The remainder of the schema is the handling of the point or spacegroup symmetry and periodic cells with three tables at the top, and *subsets* and *templates* in the lower left portion of the diagram.

**Figure 12:**
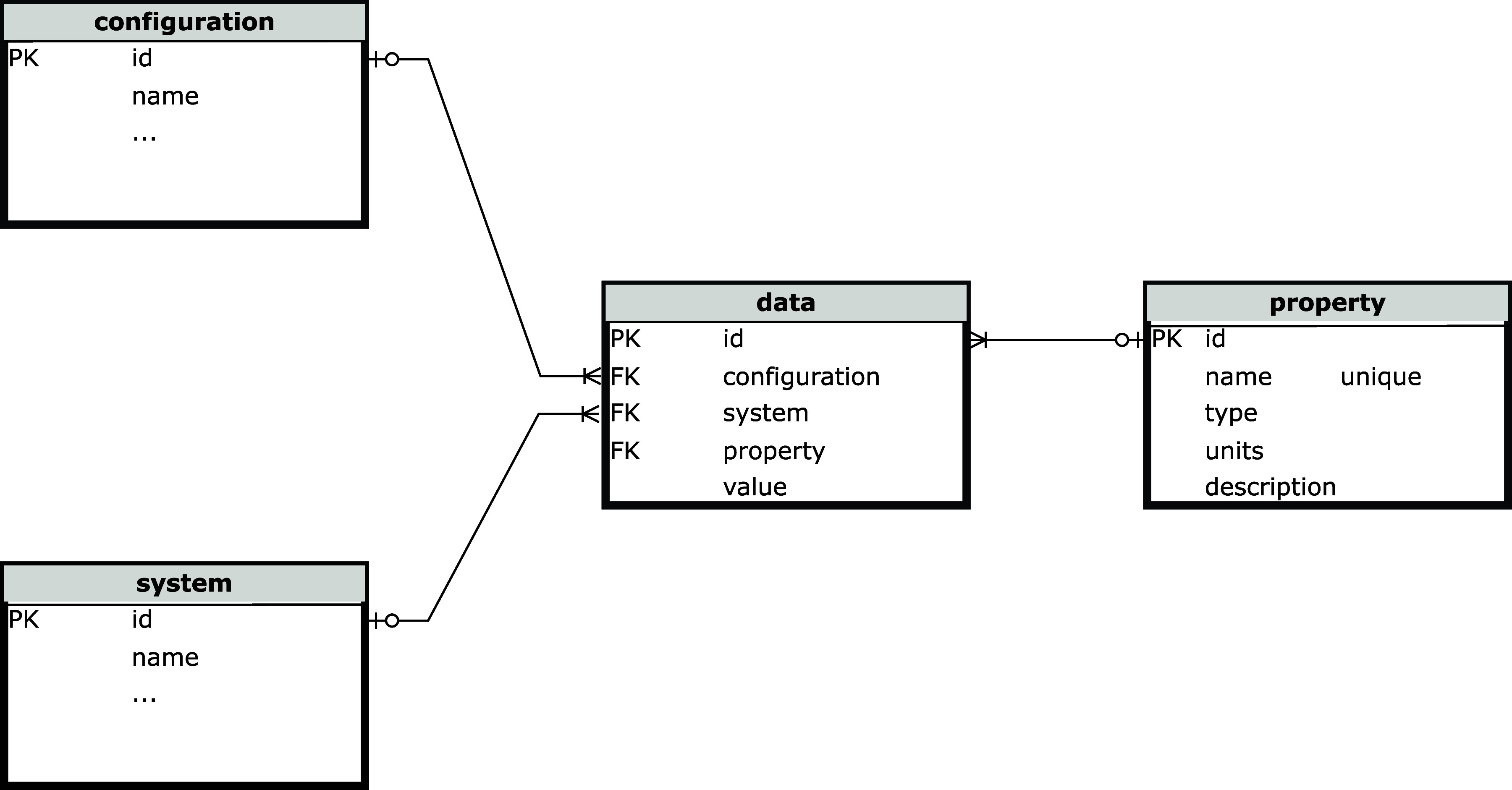
Star schema for system properties.

The second part of the schema is the star schema for handling properties and calculated results, shown in Figure 12. The values of the properties and other results are stored in the data table. In a conventional schema, the table would have a column for each property, such as energy, dipole moment, band gap, etc. This is not practical because the number of different results from all possible simulations is large but each specific simulation generates only a small number of different results. This would require an extremely wide table that would only be sparsely populated. Adding a new type of result would require changing the schema, versions of the database, *etc*., which is unsustainable.

The star schema circumvents this problem by using metadata to describe the data in the data table and storing the metadata in the property table. As such, the data table remains dense. Adding a new property or type is also possible by inserting a new row of metadata to the property table, which can be done while SEAMM is running, without the need for any change to the code requiring a new release. The disadvantage of the star schema is that finding data requires several layers of indirection or “join” operations in SQL vocabulary. Fortunately, RDBMSs are highly optimized for performing join operations because star schemas are widely used in commerce to store data, such as credit card transactions or airline reservations, which require high-performance and truly massive databases but similarly sparse data.

Similar to the part of the database that stores the structures, SEAMM provides an object-oriented facade over the star schema. The facade provides methods to define new properties, to add property data to *systems* and *configurations*, and to search for and retrieve the data. SEAMM defines standard properties such as electronic energy, formation enthalpy, dipole moment, volume or band gap, providing a *de facto* standard. The plug-ins can dynamically add any other properties that they need, such as thermal expansion coefficient, heat capacity or viscosity, though if another plug-in needs the data, the plug-ins must agree on a consistent and common naming convention.

It is impossible to overstate the importance of flexibility and extensibility of the data management systems for handling a wide range of tools and applications in the CMS fields. It is also key in ensuring SEAMM’s ability to evolve, support new codes and enable new science for many decades to come.

#### 2.2.4 Utility Library

The utility library contains a number of useful tools that can be assembled by plug-ins to meet specific needs such as

1 A comprehensive package for handling units, based on the open-source Python package Pint^78^ and customized to handle unit conversions that are common in chemistry and materials science;
2 interfaces to Open Babel,^68^ RDKit,^79^ ASE,^39^ and geomeTRIC^80^ to facilitate their use within the SEAMM environment;
3 functions to traverse the molecular graphs defined by the bonds between atoms to find individual molecules, neighbors of atoms, angles, dihedrals, and other topological features of molecules;
4 handling of the force field files; and
5 a flexible printing system modeled after the Python logging system.

#### 2.2.5 GUI Library

The GUI in SEAMM is implemented using a MVC design pattern, which allows multiple graphical implementations to be used interchangeably. The initial implementation is based on tkinter,^81^ which is Python’s native GUI package. The GUI library in SEAMM provides a number of widgets that are tailored to the needs of CMS, such as periodic tables and entry fields that have built-in unit handling. These features make the development of the GUI for SEAMM plug-ins easier and help standardize the resulting user interface and experience. There is also support for creating diagrams similar to those shown in Figure 8.

#### 2.2.6 The SEAMM Application Programming Interface for Creating Plug-Ins

Plug-ins consist of at least four components: a computational class that defines the core computational functionality of the plug-in; a graphical class that defines the GUI; a class that contains the parameters which are set by the GUI and consumed by the computational class; and a small factory class that SEAMM uses to instantiate objects of the computational and graphical classes. The first three classes are subclassed from base classes provided by SEAMM core. The factory class is minimal, consisting of two methods, each of which has a single line of code that creates and returns an instance of the compute or graphical class for the plug-in. The three base classes plus the factory class define and enforce much of the API for SEAMM. Python does not directly support virtual classes; however, base classes raise exceptions in methods that must be overridden in the subclass. These base classes also contain a number of methods that implement a significant portion of the functionality of a plug-in. In most cases, these methods are sufficient out-of-the-box, but can also be overridden for special cases.

SEAMM provides a Python Cookiecutter^82^ built on the Cookiecutter for Computational Molecular Sciences.^83^ The SEAMM Cookiecutter creates a directory and project for a new SEAMM plug-in. It is populated with the initial source files for the plug-in, a skeleton of the documentation, tests, and the files needed to create a complete project in GitHub including the GitHub Actions to check the code, run tests, and eventually release on PyPI. As such, the SEAMM Cookiecutter greatly reduces the development barrier for creating plug-ins for SEAMM and ensures their consistency with the SEAMM API. The initial skeleton, created by the SEAMM Cookiecutter can be installed and used immediately. However, it has no useful functionality on its own. Therefore, the developer’s task is to add the required parameters, edit the dialogue window if the automatic placement of the parameters is not sufficient, and create the code for executing the core function. For example, a plug-in that runs a simulation code needs to define the control parameters of the code. The GUI code will automatically display the widgets for the parameters in a table, which is sufficient if there are only a few parameters. For more complex plug-ins, the developer needs to change the code that positions the widgets and impose any dependencies between parameters if the value of one parameter affects the others. The developer must also create the code to take the parameters and create the input for the simulation code, and also reformat the atomistic structure for the code. Finally, the code to run the simulation code, and read and analyze the output also needs to be written.

The developer must supply any codes, libraries, or data that are needed to run the simulation and may need to write a simple installer for these items. SEAMM provides templates and utilities for such installers. At the very least, the plug-in must document how users can obtain and install any required code and data. If the dependencies are open-source or accessible in the public domain, the best practice is to ensure that the plug-in retrieves and installs everything that it needs. Simulation codes should be installed in separate Conda environments or Docker containers to minimize conflicts between different codes and their dependencies.

#### 2.2.7 Dashboard and Datastore

Example 1 briefly showed the Dashboard from a user’s point of view. The Dashboard is a custom web server for monitoring and managing jobs. The jobs are stored in the datastore, which is a combination of a RDBMS and files on disk. The database stores information about the jobs as well as pointers to the disk files, allowing for rapid access. The input and output of the simulations are stored as files and are indexed in the database. This allows users to directly access the files or use shell scripts and similar tools to access them if they wish. It also allows users to backup the files using tools that they are comfortable with, rather than trying to backup what would be a very large database if the simulation results were stored therein. The information in the database for each job is also saved in a disk file alongside other job files. The Dashboard can recreate the database from these files if it is corrupted.

The Dashboard provides a representational state transfer (REST) API to the datastore, allowing programmatic access to the data. The SEAMM GUI uses this API when submitting jobs to the Dashboard. Since this feature uses hypertext transfer protocol (HTTP) for data transport, SEAMM can access the Dashboard from anywhere via a browser. Users can also access the Dashboard and jobs on any machine that they can access directly or through e.g., secure shell (SSH) tunneling.

#### 2.2.8 JobServer

The JobServer is a daemon or long-running service responsible for running jobs, submitted to the Dashboard, which places them in a queue in the datastore. The JobServer accesses the datastore directly, periodically checking the queue for jobs that are waiting to run. The current version of the JobServer executes a job’s flowchart directly on the machine or in a Docker container if SEAMM is installed using Docker. Future versions will be able to work with queueing systems, such as Load Sharing Facility (LSF), which are typically used on large computer clusters.

#### 2.2.9 Flowcharts

Flowcharts are text files in JavaScript Object Notation (JSON) format that define the steps in each workflow, their control parameters, and the connections between them. Each step in a flowchart corresponds to a plug-in, represented by a graph node. The individual steps (nodes) are composed of three pieces of information defining the plug-in − the Python module of the plug-in, the class name, and the version of the plug-in − plus data that the plug-in defines and handles. This information allows a code, interpreting the flowchart, to instantiate each plug-in and have it read its internal information from the flowchart. The API for plug-ins specifies an edit method, which SEAMM uses to present the plug-in’s dialogue window to the user, and a run method, which handles the actions of the plug-in. SEAMM also provides an interpreter to execute the flowcharts. Each flowchart starts with the appropriate shebang line pointing to the interpreter. Thus, if the flowchart file is executable, it will run if called directly within a command-line interface.

This design provides a great deal of flexibility. Because the version of each plug-in is recorded in the flowchart, it is possible to use either the latest version of the plug-ins or the version specified in the flowchart. Since plug-ins are responsible for handling their own data in the flowchart, they are free to store their data in any convenient form, regardless of the aspects of data management in any other part of SEAMM. Because flowcharts are text files, they can be shared by e-mail, saved to disk, or placed in libraries. SEAMM can publish flowcharts on Zenodo,^84^ a free and open-source research repository developed by CERN and OpenAIRE, which assigns the flowcharts a digital object identifier (DOI) for future reference. Flowcharts can also be searched on Zenodo and be downloaded directly into SEAMM.

#### 2.2.10 Versioning

A major goal for SEAMM is reproducibility, and an important aspect of reproducibility is tracking the codes that are used as well as their versions. As noted above, flowcharts are an important tool for reproducibility, and they track internally the versions of the plug-ins used. The SEAMM installer also tracks the modifications to the SEAMM environment it makes, saving a machine-readable description of the environment in time-stamped files after each modification. These can be used to recreate the environment as it was at any given time. This tracking is described in more detail in the Supporting Information.

## 3 Results and Discussion

### 3.1 Example 2: Rearrangement of Methylisocyanide to Acetonitrile

One of the strengths of SEAMM is its ability to use a range of codes to tackle complex multiscale problems in CMS. This section will focus on the rearrangement of methylisocyanide to acetonitrile, which is a prototype for unimolecular reactions and has been studied extensively, both experimentally and computationally.^85–89^ We will reproduce the results of one of the first computational studies^88^ and then replicate the results using a wide range of computational methods in SEAMM. The focus will be on the energy of the transition state structure and the curvature of the potential energy surface at the saddle point.

We will start by reproducing the optimized transition state structure and its harmonic vibrational frequencies as provided in ref. 88 and then move to locate the transition state structure from scratch. Given the structure, it is straightforward to calculate the vibrational frequencies and normal modes using quantum chemistry software such as Gaussian^10^ or Psi4.^90^ The results are shown in Table 1.

**Table 1:** Vibrational Frequencies (cm^−1^) and Energies (*E*_h_) of the Transition-State Structure for the Rearrangement of Methylisocyanide to Acetonitrile Calculated at the HF/DZP Level of Theory^a^

	**Ref.** ^88^	**G** **aussian**	**P** **si** **4**	**Finite Difference**
Frequencies (cm^−1^)	458*i*	458*i*	458*i*	459*i*
	255	255	255	255
	677	677	677	677
	1063	1063	1063	1063
	1083	1083	1083	1083
	1457	1457	1457	1456
	1590	1590	1590	1589
	1599	1599	1599	1599
	2189	2189	2189	2189
	3272	3272	3272	3272
	3388	3388	3388	3388
	3411	3411	3411	3411
Energy (*E*_**h**_)	−131.84756	−131.84744	−131.84738	−131.84744

a The reference values are obtained from Ref. 88.

The first column lists the reference frequencies obtained from ref. 88, where the second derivatives needed for the harmonic vibrational analysis were calculated using finite differences of gradients. The second and third columns of Table 1 show the vibrational frequencies calculated using Gaussian and Psi4, respectively, using analytic second derivatives. The analytic frequencies are identical to those of ref. 88 within less than 1 cm^−1^ while the energies are in perfect agreement within 0.0002 *E*_h_ (or ≈ 0.13 kcal mol^−1^). The different codes make different approximations to reduce the computational cost. For example, Psi4 uses the resolution of identity (RI) and various cutoffs by default when evaluating the two-center integrals. Gaussian, on the other hand, does not use RI. As noted in ref. 88, the code used at the time could not handle the *d* functions in the basis set. As such, the *d* functions were approximated by off-center *p* orbitals which introduced an error of ≈0.0001 *E*_h_. Note that such errors or approximations tend to be systematic. Thus, relative energies are more meaningful and accurate in this context. In this section, we have made no effort to tune the input parameters in Psi4 and Gaussian and benefited from reasonable default values in SEAMM to reduce errors from the approximations made to gain performance. The precision of the current results is also well within chemical accuracy and on-par with the precision of the original calculations performed more than 40 years ago. These results provide strong evidence for the reproducibility of the quantum chemical calculations between different codes with quite different implementations and running on very different computers over a span of decades.

Both Gaussian and Psi4, as well as a number of other codes, with available interfaces in SEAMM, can calculate second derivatives analytically. However, many codes may not offer this feature. The Thermochemistry plug-in in SEAMM eliminates this issue by using finite differences or the first derivatives to calculate harmonic vibrational frequencies and properties. The last column in Table 1 shows the results of the finite difference approach mentioned above using Gaussian to calculate the energy and first derivatives. Using the default step size of 0.01 Å in the Thermochemistry plug-in, the results are essentially identical to those calculated analytically, with a deviation of 1 cm^−1^ in the frequencies. An advantage of using the Thermochemistry plug-in is that it ensures that the vibrational analysis is handled identically when using different methods and codes. As an added benefit, the Thermochemistry plug-in makes it easy to switch between different simulation codes, since only a small subflowchart, responsible for calculating the energy and forces, needs to be changed.

Beyond reproducing the results for the given transition state structure, the Reaction Path plug-in for SEAMM finds approximate reaction paths and transition states using the nudged elastic band (NEB) method.^91–95^ Once an approximate transition state is located, the Structure plug-in can optimize it, in the process ensuring that there is a single mode with an imaginary frequency. Similar to the Thermochemistry plug-in, both plug-ins only need the energy and gradients of the structure, allowing them to be used with a wide range of simulation methods in SEAMM.

Table 2 shows the relative enthalpies of the structures in the reaction path, as well as the imaginary frequency of the transition state, calculated with a range of codes and methods, ranging from the *ab initio* Hartree−Fock method with Gaussian or numerical atomic orbitals, semiempirical Hartree−Fock methods in MOPAC, and semiempirical DFT using DFTB+. The *ab initio* Hartree−Fock methods give almost identical results. The observed small differences are mainly due to differences in the quality of the analytical and numerical basis sets in various codes. The results presented in Table 2 demonstrate that semiempirical methods, whether based on Hartree−Fock or DFT, overestimate the reaction energy barrier height and have a larger variance for both the enthalpy of reaction and the imaginary frequency.

**Table 2:** Comparison of the Enthalpies (kcal mol^−1^ at 298 K and 1atm) of the Structures of Methylisocyanide, the Transition State, and Acetonitrile Relative to Methylisocyanide, Plus the Curvature of the Imaginary Mode at the Transition State for a Range of Different Models

	**HF/DZP** ^a^	**HF/tight** ^b^	**PM6-org** ^c^	**PM7** ^d^	**DFTB/3ob** ^e^	**xTB/GFN2** ^f^
**CH**_**3**_ **NC**	0.0	0.0	0.0	0.0	0.0	0.0
**TS**	43.6	42.5	60.5	73.1	60.2	62.8
**CH**_**3**_ **NC**	−19.5	−20.0	−29.5	−20.7	−10.4	−23.5
**Freq** ^g^	458.0*i*	463.0*i*	577.0*i*	696.0*i*	343.0*i*	412.0*i*

a Hartree−Fock with Gaussian using the DZP basis.^96^

b Hartree−Fock with FHI-aims^97,98^ using the tight basis.^99^

c Hartree−Fock with MOPAC^100^ using the PM6-ORG parametrization.^101^

d Hartree−Fock with MOPAC using the PM7 parametrization.^102^

e DFTB^103^ with DFTB+^104^ using the 3ob parametrization.^105^

f xTB^106^ with DFTB+ using the GFN2 parametrization.^107^

g Frequencies are in cm^−1^.

Similar calculations with ReaxFF force fields^108–110^ and machine learning potentials^111,112^ could not locate the transition state, nor could the transition state be successfully optimized starting from the known transition state structure using these force fields or potentials. Although details of the parametrization are difficult to track in these methods, it appears that the training data for all of the methods examined include cyanides but not isocyanides. Each of the potentials tried predicts a nonlinear C−N−C bond angle in methylisocyanide instead of the known linear structure, indicating that the extrapolation of the potentials to the isocyanide functional group is not reasonable. So, while SEAMM is capable of locating the transition state and calculating its thermochemistry using such potentials, for this particular reaction none of the force fields or machine learning potentials tried in this study could represent the potential energy surface in a physically reasonable way, because the reactant and transition state are too far from the training sets. More details about the calculations and results in this example are included in the Supporting Information, along with a description of the flowchart used.

### 3.2 Example 3: Advancing Understanding of Battery Materials

SEAMM is not only a productivity software for reproducible CMS workflows and a powerful engine for academic research, but it is also a valuable tool for industrial research. In this example, we highlight a real-world application of SEAMM in predicting the transport properties of lithium ion battery materials via MD simulations. This study has two main parts. First, we will discuss the simulation of transport properties such as ionic diffusivity, conductivity, and transference number in liquid electrolytes composed of ethylene carbonate (EC), ethyl methyl carbonate (EMC), and dimethyl carbonate (DMC), such as EC:EMC (3:7 w/w) and EC:DMC (1:1 w/w) with lithium hexafluorophosphate (LiPF_6_) at room temperature. Second, we will discuss the thermal expansion in battery electrodes such as LiCoO_2_ at finite temperature and their thermal conductivity as a function of the state of lithiation (SOL) at room temperature.

The performance of lithium-ion batteries at low temperatures or under rapid charge−discharge conditions is governed by the intrinsic transport properties of their liquid electrolytes. Despite their importance, even for conventional electrolytes, the available transport measurement data at various concentrations and temperatures are scarce in the literature. An alternative to measurement techniques is to simulate the system in order to predict the desired transport properties under various operating conditions that are not otherwise easily accessible through experiments. Here, we demonstrate the application of classical MD via the LAMMPS code to model transport in EC:EMC (3:7 w/w) and EC:DMC (1:1 w/w) electrolytes with various concentrations of LiPF_6_ salt at room temperature. The simulations were performed using SEAMM flowcharts to build the MD input files, call the LAMMPS calculator, and finally use custom Python scripts for postprocessing of the trajectory data.

Table 3 shows the SMILES of different solvents and the salt used in our simulations which are EC, EMC, DMC, and LiPF_6_ salt. The initial MD configuration is generated using the PACKMOL step, which reads the SMILES strings of the solvents and salt based on the given electrolyte formulation and creates a cubic periodic cell to initiate the LAMMPS simulation. The OPLS-AA force field^56,113^ was used to describe the bonded and nonbonded parameters of each solvent. The parameters used for the Li^+^ cations and LiPF_6_ anions were from Jensen et al.^114^ and Canongia Lopes and Pádua,^115^ respectively.

**Table 3:** Solvent and Salt SMILES Strings, Experimental Density and MD Simulation Density at 25 °C

**Solvent/Salt**	**SMILES**	**Exp. Density** **(g cm** ^**−3**^ **)**	**MD Density** **(g cm** ^**−3**^ **)**
**EC**	C1COC(=O)O1	1.32	1.33
**EMC**	CCOC(=O)OC	1.01	1.03
**DMC**	COC(=O)OC	1.07	1.08
**LiPF**_**6**_	[Li^+^]·F[P^−^](F)(F)(F)(F)F	-	-

The individual density of each solvent was first validated through MD simulations, as shown in Table 3. These densities were used to make an initial guess for the density and hence dimensions of the initial configuration of the electrolyte formulations. The LAMMPS simulation involved a structural minimization followed by 2 ns NPT canonical dynamics using a Nose−Hoover thermostat and barostat at 25 °C and 1 atm to equilibrate the system. The cell was then adjusted isotropically to the equilibrium density and the results of 5 production runs of 200 ps of NVT dynamics were averaged. Using SEAMM’s Diffusivity module, the position and velocity trajectories were analyzed and the diffusion constant was calculated using the MSD approach. The slope of the linear regime in the MSD was obtained for each simulation of 200 ps duration and averaged over the total of 1 ns of the production runs to obtain the diffusion constant, defined as^116,117^

1 $$D\left(T\right)=\frac{\left\langle \mathrm{MSD}\left(T\right)\right\rangle }{6\Delta t}$$

The electrolyte salt diffusivity, $D_{{\mathrm{LiPF}}_{6}}\left(T\right)$ , was calculated from the individual diffusivities of Li^+^ cations and PF_6_^−^ anions in the cell as^118^

2 $$D_{{\mathrm{LiPF}}_{6}}\left(T\right)=\frac{2D_{{\mathrm{Li}}^{+}}\left(T\right)D_{{{\mathrm{PF}}_{6}}^{-}}\left(T\right)}{D_{{\mathrm{Li}}^{+}}\left(T\right)+D_{{{\mathrm{PF}}_{6}}^{-}}\left(T\right)}$$

The electrolyte ionic conductivity, σ­(*T*), can also be calculated using the Nernst−Einstein equation,^119^

3 $$\sigma \left(T\right)=\frac{N_{\mathrm{Li}}e^{2}}{Vk_{B}T}\left(D_{{\mathrm{Li}}^{+}}\left(T\right)+D_{{{\mathrm{PF}}_{6}}^{-}}\left(T\right)\right)$$

where *N*_Li_ is the number of Li^+^ ions and *V* is the volume of the simulation cell, *e* is the electronic charge, *T* is the temperature, $D_{{\mathrm{Li}}^{+}}$ is the Li^+^ cation diffusion constant, and $D_{{{\mathrm{PF}}_{6}}^{-}}$ is the PF_6_^−^ anion diffusion constant. The cationic transference is expressed in terms of the ratios of the ionic diffusivities,^120^

4 $$t_{+}=\frac{D_{{\mathrm{Li}}^{+}}}{D_{{\mathrm{Li}}^{+}}+D_{{{\mathrm{PF}}_{6}}^{-}}}$$

**Figure 13:**
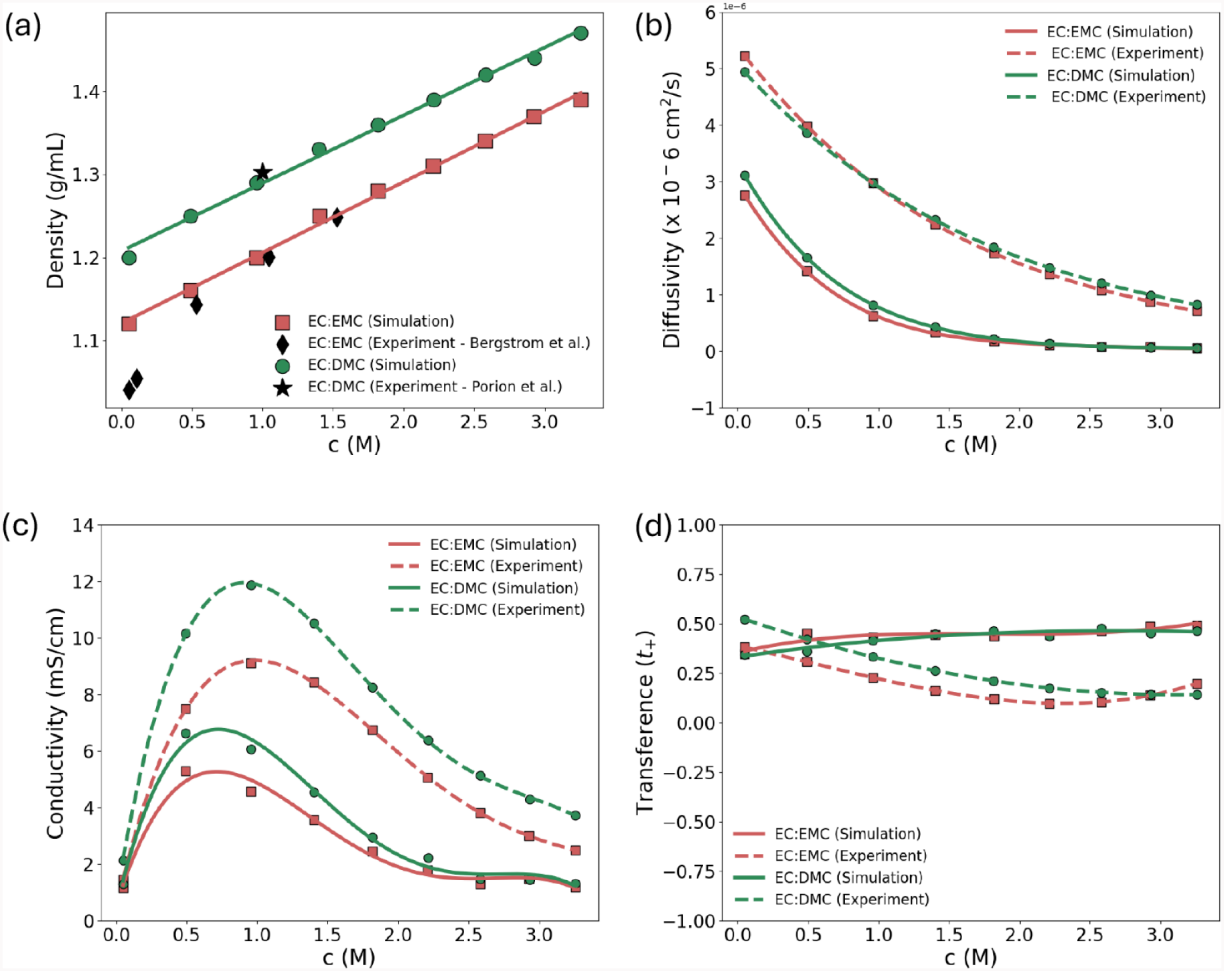
Simulation of transport properties of lithium ion battery liquid electrolytes, EC:EMC (3:7 w/w) and EC:DMC (1:1 w/w), at various molar concentrations (*c*) of LiPF_6_ salt at 25 °C. Panels (a−d) show the MD density, ionic diffusivity, ionic conductivity, and cationic transference number, respectively, which are obtained from MD simulation and compared with experimental results performed at 25 °C. The experimental densities in (a) were taken from refs. 121 and 122. The experimental results for diffusivity, conductivity and transference numbers in (b−d) were taken from ref. 123.

Figure 13 shows the calculated transport properties from MD simulations of EC:EMC (3:7 w/w) and EC:DMC (1:1 w/w) electrolytes, at various molar concentrations (*c*) of LiPF_6_ salt at 25 °C. The equilibrium densities [Figure 13a], obtained from the MD simulation, are in agreement with the experimental mass densities of EC:EMC (3:7 w/w)^121^ and EC:DMC (1:1 w/w)^122^ electrolytes, especially at a salt concentration >0.5 M. At any salt concentration, the EC:DMC (1:1 w/w) mixture resulted in a higher density values than those of EC:EMC (3:7 w/w). Landesfeind and Gasteiger^123^ performed an extensive experimental study on the temperature and concentration dependence of the ionic transport properties of these electrolytes. We compare the simulated ionic diffusivity, conductivity and transference number with their result as shown in Figure 13b−d. We found that the predicted ionic transport properties from the simulation underestimated the experimental results, which could be attributed to the default ionic charges of the OPLS-AA force-fields. It has been reported in earlier literature that the transport properties in high-concentration electrolytes can be significantly underestimated if the ionic charge correction is neglected^124,125^ in the MD simulations conducted with OPLS-AA force-fields. Even using the recently improved OPLS4 force-field parameters,^126^ the predicted values of the transport and thermodynamic properties of various ions and solvated molecules of common ionic liquids for a wide range of temperatures^127^ were underestimated. However, even though the relative values of the ionic properties are underestimated in the simulations, the overall trends in the experimental property values between electrolyte formulations are accurately conserved within the MD simulations. Both experiment and simulation results demonstrate that both electrolyte formulations in our study have similar ionic diffusions. Nonetheless, the EC:DMC (1:1 w/w) mixture exhibited much higher ionic conductivity and cationic transference number than those of EC:EMC (3:7 w/w), due to its higher mass density.

**Figure 14:**
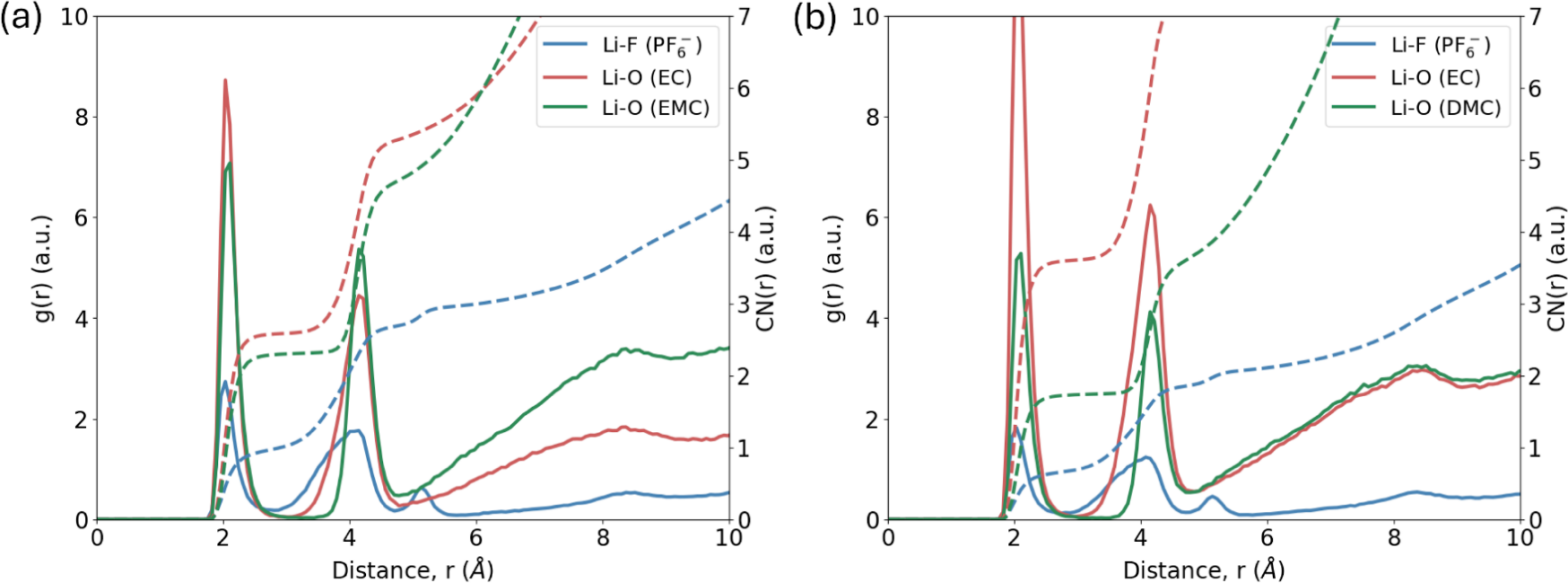
Calculated radial distribution function, *g*(*r*), and corresponding coordination number integrals, CN­(*r*), of (a) Li−F (PF_6_^−^), Li−O (EC), Li−O (EMC) in EC:EMC (3:7 w/w) with 1 M LiPF_6_, and (b) Li−F (PF_6_^−^), Li−O (EC), Li−O (DMC) in EC:DMC (1:1 w/w) with 1 M LiPF_6_.

One of the key functionalities of SEAMM workflows is the ability to write custom python scripts within flowcharts, which enable on-the-fly postprocessing of the MD simulation data. Figure 14 shows one such example where the radial distribution function, *g*(*r*), and the corresponding coordination number, CN­(*r*), of solvated Li^+^ ions in the above electrolyte formulations were calculated using a custom script that analyzed the MD trajectory data. In both formulations, we found that the first solvation shell is mostly occupied by the EC solvent molecules with a most probable distance of 2.05 Å to Li^+^, which is in agreement with previous MD studies of similar electrolyte systems.^128^ The presence of EMC and DMC molecules within the first solvation shell depends on their relative ratios to the EC solvent molecules. We found a substantial presence of EMC in EC:EMC (3:7 w/w) while the presence of DMC was almost half of EC in EC:DMC (1:1 w/w). The average Li^+^ ion coordination number with solvent and salt is 5.84 for EC:EMC (3:7 w/w) and 5.98 for EC:DMC (1:1 w/w), which are also in good agreement with earlier MD studies.^128^

**Figure 15:**
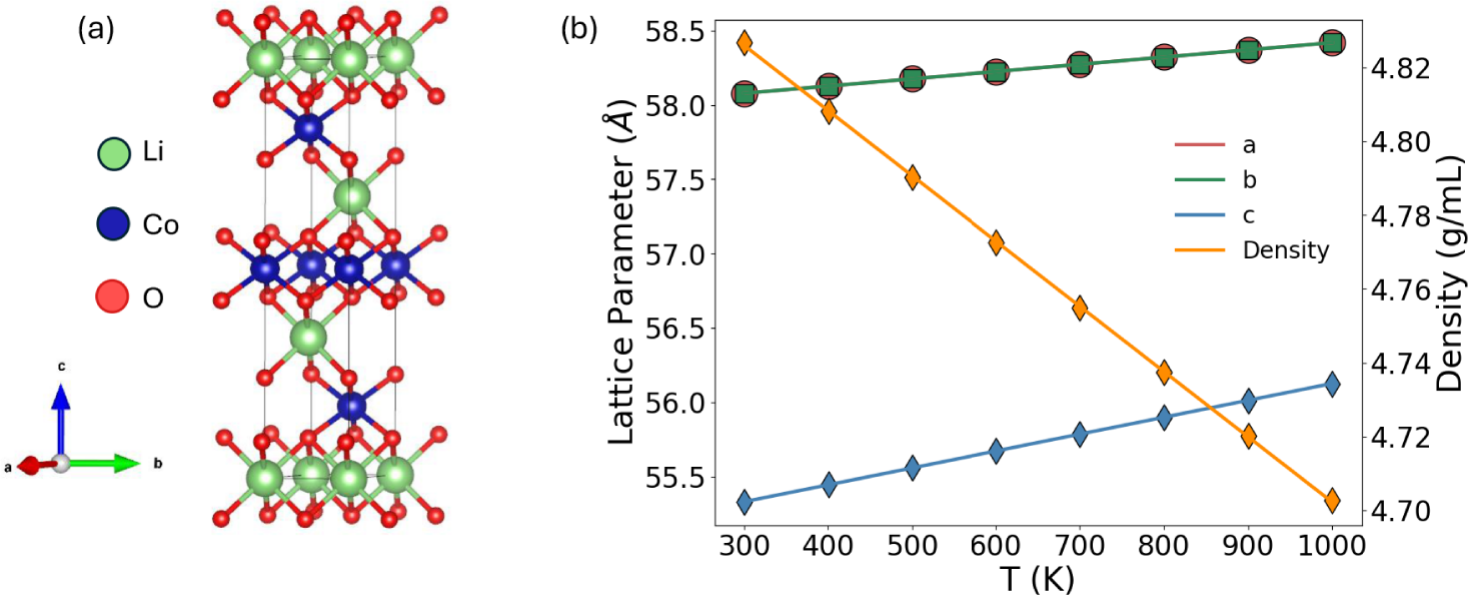
Thermal expansion in LiCoO_2_. (a) The experimental layered (*R*3*m*) crystal structure of LiCoO_2_ used in the MD simulation. The Li, Co and O atoms are represented as green, blue and red spheres. (b) Lattice parameter expansion and change in density as a function of temperature in LiCoO_2_.

**Figure 16:**
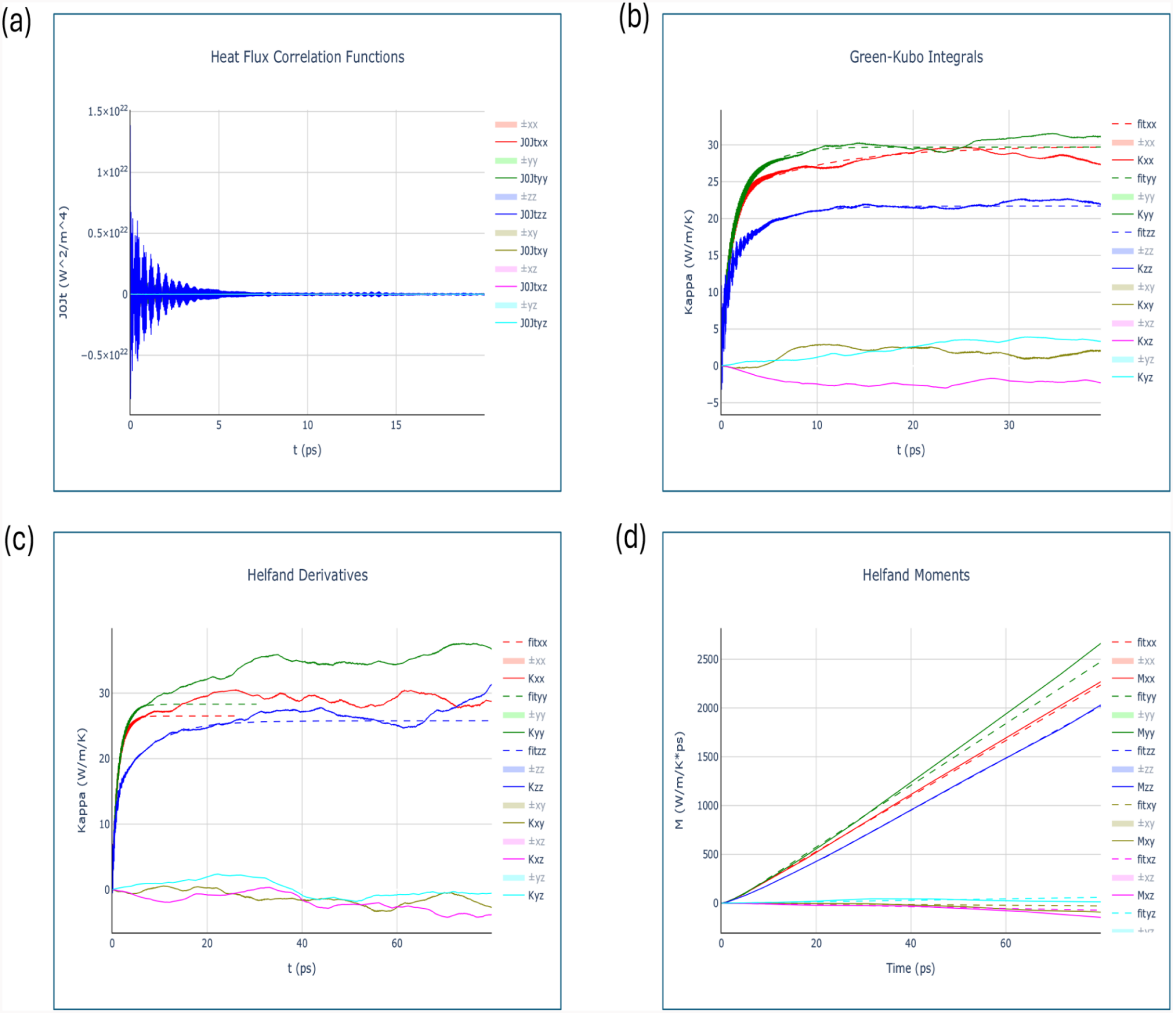
Thermal conductivity simulation of LiCoO_2_ using equilibrium MD at *T* = 298 K. Sample calculation of (a) heat flux autocorrelation function, (b) thermal conductivity fittings of Green−Kubo integrals, (c) thermal conductivity fitting of Helfand derivatives, and (d) Helfand moments as a function of time in ps.

**Figure 17:**
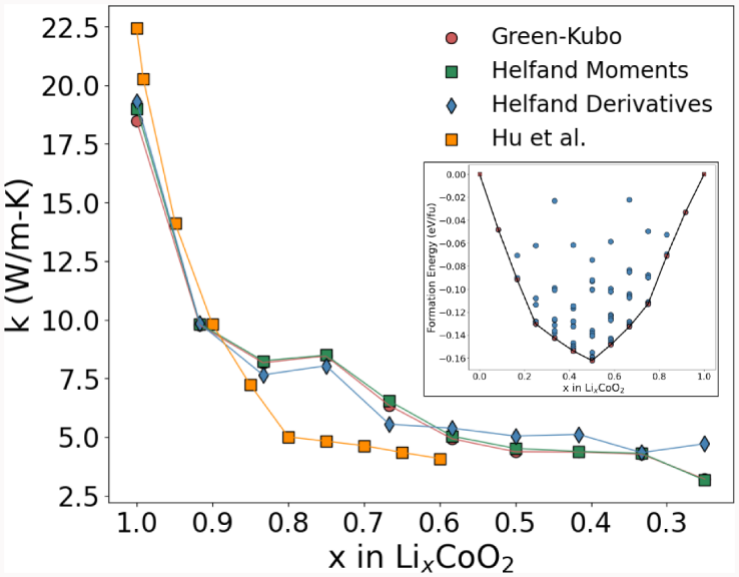
Calculated thermal conductivity, k (W/m K) of Li_*x*_CoO_2_ as a function of Li concentrations at *T* = 298 K from the MD simulation. The inset shows a convex hull diagram calculated using ab initio ground state formation energies to predict the lowest energy Li vacancy structures.

In the next part of our study, we focus on the prediction of the thermal expansion of LiCoO_2_ at finite temperature using LAMMPS, as well as the simulation of thermal conductivity at various SOL at room temperature. We used SEAMM flowcharts for each step of the equilibrium MD simulations. Crystalline layered LiCoO_2_ with rhombohedral symmetry (space group *R*3*m*, as shown in Figure 15a^129^), and the Buckingham potential^130^ developed by Fisher et al.^131^ were used for the simulations. The potential energy between pairs of ions in each crystalline solid was calculated by combining the long-range Coulombic component with a short-range repulsive and attractive van der Waals interaction as described in ref. 131. The short-range interactions, ϕ_*i,j*_, were described by a Buckingham potential of the form^131^

5 $$\phi_{i,j}\left(r_{ij}\right)=A_{ij}\exp\left(-r_{ij}/\rho_{ij}\right)-C_{ij}/r_{ij}^{6}$$

where, *r*_*ij*_ is the distance between the ions *i* and *j*, *A*_*ij*_, ρ_*ij*_ and *C*_*ij*_ are parameters fit to experimental lattice parameters and ion positions as described in ref. 131. A cutoff distance of 14 Å was used for the Coulombic and Buckingham terms. First, equilibrium MD simulations with the isothermal−isobaric (NPT) ensemble were performed using a Nose−Hoover thermostat and barostat at a temperature *T* and a pressure of 1 atm for 200 ps, using a time step of 1 fs, to achieve thermal equilibration and to relax the structure. The lattice parameters of the relaxed geometry at each temperature *T* were then collected for the expansion calculation.

Figure 15b shows the in-plane and out-of-plane lattice parameters and the change in mass density in LiCoO_2_ as a function of temperature. The corresponding expansion coefficients (defined as Δ*L*/*L*Δ*T*, where *L* is the length of a lattice constant) along the lattice direction of *a*, *b* and *c* are shown in Table 4. The in-plane expansions are identical for the *a* and *b* directions and much smaller than the expansion along the out-of-plane *c* direction. This anisotropic lattice expansion is expected since the LiCoO_2_ has a layered crystal structure where the Co d orbitals have strong Coulomb interactions with the in-plane oxygen 2p orbitals^132^ and the interlayer interaction is a van der Waals interaction between the adjacent layers along the *c*-axis.^133^ The average thermal expansion coefficient predicted by the MD simulation (1.24 × 10^-5^ K^−1^) is in close agreement with the experimental expansion coefficient from the polycrystalline LiCoO_2_ pellets (1.3 × 10^−5^ K^−1^).^134^

**Table 4:** Thermal Expansion in LiCoO_2_

**Lattice Constant**	**Expansion (K** ^**−1**^ **)**
*a*	8.36 × 10^−6^
*b*	8.36 × 10^−6^
*c*	2.04 × 10^−5^
**Average Expansion**	1.24 × 10^−5^
**Experimental Expansion**	1.30 × 10^−5^

Equilibrium MD methods such as Green−Kubo formalism,^135,136^ Helfand derivatives, and Helfand moments^137–139^ were used to predict the thermal conductivity of LiCoO_2_. According to the Green−Kubo theory, which is derived from the fluctuation dissipation theorem and linear response theory, the thermal conductivity tensor κ^αβ^ is proportional to the time integral of the heat flux autocorrelation function,^135,136^

6 $$\kappa^{\alpha \beta }\left(T\right)=\frac{1}{k_{B}T^{2}V}\int_{0}^{\infty }\left\langle J^{\alpha }\left(0\right)\cdot J^{\beta }\left(t\right)\right\rangle dt$$

where *J* ^α^(*t*) is the αth Cartesian component of the time-dependent heat current, *k*_B_ is the Boltzmann constant, *V* is the system volume, *T* is the system temperature, and ⟨·⟩denotes the ensemble average. In addition to the Green−Kubo approach, the Helfand moment and Helfand derivative approaches, which follow directly from the Green−Kubo expression^137–139^ were used to calculate the thermal conductivity. The Helfand moment *G* ^α^(*t*) is defined in terms of the time integral of the heat current component *J* ^α^ as

7 $$G^{\alpha }\left(t\right)=G^{\alpha }\left(0\right)+\int_{0}^{t}J^{\alpha }\left(\tau \right)d\tau$$

in which, the ensemble-averaged quantity, *M* ^αβ^, is defined as^138^

8 $$M^{\alpha \beta }\left(t\right)=\left\langle \left(G^{\alpha }\left(t\right)-G^{\alpha }\left(0\right)\right)\left(G^{\beta }\left(t\right)-G^{\beta }\left(0\right)\right)\right\rangle$$

For a sufficiently long simulation time, *M* ^αβ^(*t*) grows linearly as a function of time with a slope that is directly proportional to the corresponding thermal conductivity tensor component κ^αβ^(*T*).^140^ Therefore, the thermal conductivity tensor κ^αβ^(*T*) is then defined in the Helfand approach as

9 $$\kappa^{\alpha \beta }\left(T\right)=\frac{1}{2tk_{B}T^{2}V}\underset{t\to \infty }{\lim}M^{\alpha \beta }\left(t\right)$$

The advantage of the Helfand method over the more traditional Green−Kubo approach is 2-fold. First, its convergence with simulation time is superior to that of Green−Kubo. Second, it is easier to fit the straight line given by eq 9 than the exponential rise of the Green−Kubo function, which is complicated by the increasing noise as a function of time.

In order to calculate the time-dependent heat current autocorrelation function and apply it to the thermal conductivity calculation using the Green−Kubo and Helfand approaches, we performed production MD simulation with various run times. Before running the production runs, the system was further equilibrated for 200 ps, with a time step of 1 fs, of canonical (NVT) dynamics at a temperature *T* using a Nose−Hoover thermostat. Then simulations using the microcanonical (NVE) ensemble with a run length of 200 ps, 400 ps and 800 ps were carried out 10 times for each run length, for a total of 30 production runs, to calculate the heat flux autocorrelation function. The results of all the production runs were averaged to give the final thermal conductivity. Figure 16 shows a sample calculation of heat flux autocorrelation function, the Green−Kubo integrals, Helfand derivative, and Helfand moments for fully lithiated LiCoO_2_ at *T* = 300 K for a 400 ps production run.

Figure 17 shows the calculated thermal conductivity of LiCoO_2_ at various lithium concentrations at temperature of *T* = 298 K. Each Li vacancy-ordered structure was first generated using DFT to calculate the formation energy of the convex hull of the ground state (*T* = 0 K). The inset in Figure 17 shows the convex hull diagram where the lowest energy structure was considered to be the most thermodynamically stable structure at Li concentration *x*. Once these structures were identified, we used the classical MD simulation described above to calculate the thermal conductivity at *T* = 298 K. All three approaches (Green−Kubo, Helfand moments, and Helfand derivatives) resulted in very similar thermal conductivities at any *x* considered in our calculations. Our results indicate that with the decrease in Li concentration during battery charging, the thermal conductivity decreases drastically from about 20 W/m/K at *x* = 1 to 3.2 W/m/K at *x* = 0.3. These results agree quantitatively with an earlier MD study by Hu et al.^141^ where the nonequilibrium MD was used for the prediction of the thermal conductivity in LiCoO_2_.

## 4 Conclusions

We have presented SEAMM, a new open source productivity tool and workflow environment for computational modeling and simulations of molecular and solid-state materials. SEAMM is underpinned by an extensive data model for molecular and crystalline structures implemented in an in-memory relational database using a traditional schema. The database also stores calculated properties and results by describing them with metadata using a star schema to connect the data to the metadata, allowing the data model to be modified and extended on the fly. This flexibility will permit SEAMM to adapt and change as current simulation tools evolve and new tools and methods are developed. An object-oriented facade is layered on top of the database, giving SEAMM a conventional data model from the software developers point of view. However, the flexibility and extensibility of the underlying model is carried through to the object-oriented model.

The heart of SEAMM are flowcharts, which represent simulation workflows in a graphical form. The functionality that users see in SEAMM is provided by independent software modules that plug into SEAMM and share data through the in-memory database. Each plug-in corresponds to a step in a flowchart, presents its own graphical interface to users, implements the needed computation or simulation, and analyzes and presents the results. The only connection between plug-ins is the underlying data model in SEAMM. This decentralized approach allows any number of different groups to develop plug-ins with little or no coordination between groups. Users then create flowcharts to implement their workflows, combining plug-ins to do the simulations and modeling that are needed for their scientific or engineering problem.

There are a number of scriptable workflow tools for CMS, as well as a number of graphical tools for specific tasks. However, there is no general open source graphical workflow system for CMS. SEAMM fills this gap and extends the range of CMS tools. With a focus on productivity, on reducing the entry barrier to using a wide range of CMS simulation tools, and on the reproducibility and transparency of the workflows, SEAMM is a valuable contribution to the community.

## Supplementary Material



## Data Availability

The source code for SEAMM is openly available in GitHub at https://github.com/molssi-seamm. The data associated with Examples 1 and 2 is openly available in Zenodo at 10.5281/zenodo.15352015

## Notes

Simulations details and the results of Example 2 in the paper in more detail, calculation results underlying Tables 1 and 2, expanded computational results with ReaxFF and TorchaANI alongside the exploration of the potential energy surfaces for the aforementioned methods; description of the versioning in SEAMM and associated files containing version information (ZIP)

## Abbreviations

API: application programming interface
AQM: automated quantum mechanical environments for researchers and educators
ASE: atomic simulation environment
AC: autocorrelation function
BSE: basis set exchange
CI-NEB: climbing image nudged elastic band
CMS: computational molecular science
DOI: digital object identifier
DFT: density functional theory
DMC: dimethyl carbonate
EC: ethylene carbonate
EMC: ethyl methyl carbonate
GIMS: graphical interface for materials simulations
GUI: graphical user interface
IDPP: image-dependent pair potential
JSON: JavaScript Object Notation
LAMMPS: large-scale atomic/molecular massively parallel simulator
MD: molecular dynamics
MSD: mean square displacement
MVC: model-view-controller
NEB: nudged elastic band
OPLS-AA: optimized potentials for liquid simulations − all atom
PyPI: the Python Package Index
RDBMS: relational database management system
REST: representational state transfer
RI: resolution of identity
SEAMMS: simulation environment for atomistic and molecular modeling
SDF: structure-data format
SMARTS: SMILES arbitrary target specification
SMILES: simplified molecular input line entry system
SOL: state of lithiation
SQL: structured query language
VASP: Vienna Ab initio Simulation Package
