# Supplementary material for: SEAMM: A Simulation Environment for Atomistic and Molecular Modeling
Source: J Phys Chem A. 2025 Jul 18;129(30):6973–93. doi: 10.1021/acs.jpca.5c03164 (PMC12319917; doi:10.1021/acs.jpca.5c03164)
Supplement: Supplementary file 1 [file jp5c03164_si_001.pdf]

Supporting Information: SEAMM: A Simulation Environment for Atomistic and Molecular Modeling

Paul Saxe,[*,†,‡] Jessica Nash,[*,†,‡] Mohammad Mostafanejad,[*,†,‡] Eliseo Marin-Rimoldi,[*,¶] Hasnain Hafiz,[*,§] Louis G Hector, Jr.,[*,§] and T. Daniel Crawford[*,†,‡]

†*Department of Chemistry, Virginia Tech, Blacksburg, Virginia 24061, USA*

‡*Molecular Sciences Software Institute, Blacksburg, Virginia 24060, USA*

¶*Department of Chemical and Biomolecular Engineering, University of Notre Dame, Notre Dame, Indiana 46556, USA*

§*Battery Research and Development, General Motors, Warren, MI 48092, USA*

E-mail: psaxe@vt.edu; janash@vt.edu; smostafanejad@vt.edu; emarinri@nd.edu; hasnain.hafiz@gm.com; louis.hector@gm.com; crawdad@vt.edu

Contents

Introduction **3**

Flowchart for Reaction Paths **3**

Initialization 5

Optimization of the Reactant and Product Structures 6

The Reaction Path and Approximate Transition States 7

Optimization of the Key Structures and Their Thermochemistry 8

Reaction Path for the Rearrangement of Methylisocyanide to Isonitrile **11**

Components of SEAMM **14**

Understanding versioning in SEAMM **15**

Introduction

This supplementary material covers the simulations and results of Example 2 in the paper in more detail. The first part describes the calculations underlying Tables 1 and 2. The second part covers the calculations with ReaxFF and TorchANI, expanding on the results mentioned in the paper and some exploration of the nature of the potential energy surface for these methods.

Flowchart for Reaction Paths

Finding the transition state for a reaction and calculating the energies and enthalpies of the reactants, transition state, and products requires a number of distinct calculations:

1. Determining the structures of the reactant(s) and product(s);
2. Locating the transition state between the reactants(s) and product(s);
3. Refining the structure of the transition state;
4. Calculating the thermochemical functions in the harmonic approximation for the reactant(s), transition state, and product(s)

Each of these parts can be handled with a separate flowchart in a Simulation Environment for Atomistic and Molecular Modeling (SEAMM), or two or more parts can be combined in a single flowchart. Figure 1 shows a single flowchart for handling all the needed calculations, which is convenient for running either many different methods for a single reaction, or investigating many reactions.

This flowchart corresponds directly to the steps above. The next subsections describe the flowchart in some detail because it nicely illustrates the flexibility and power of SEAMM. However, since the flowchart implements a reasonably complicated workflow, the description is quite lengthy, and may be skipped if the detail is not of interest.

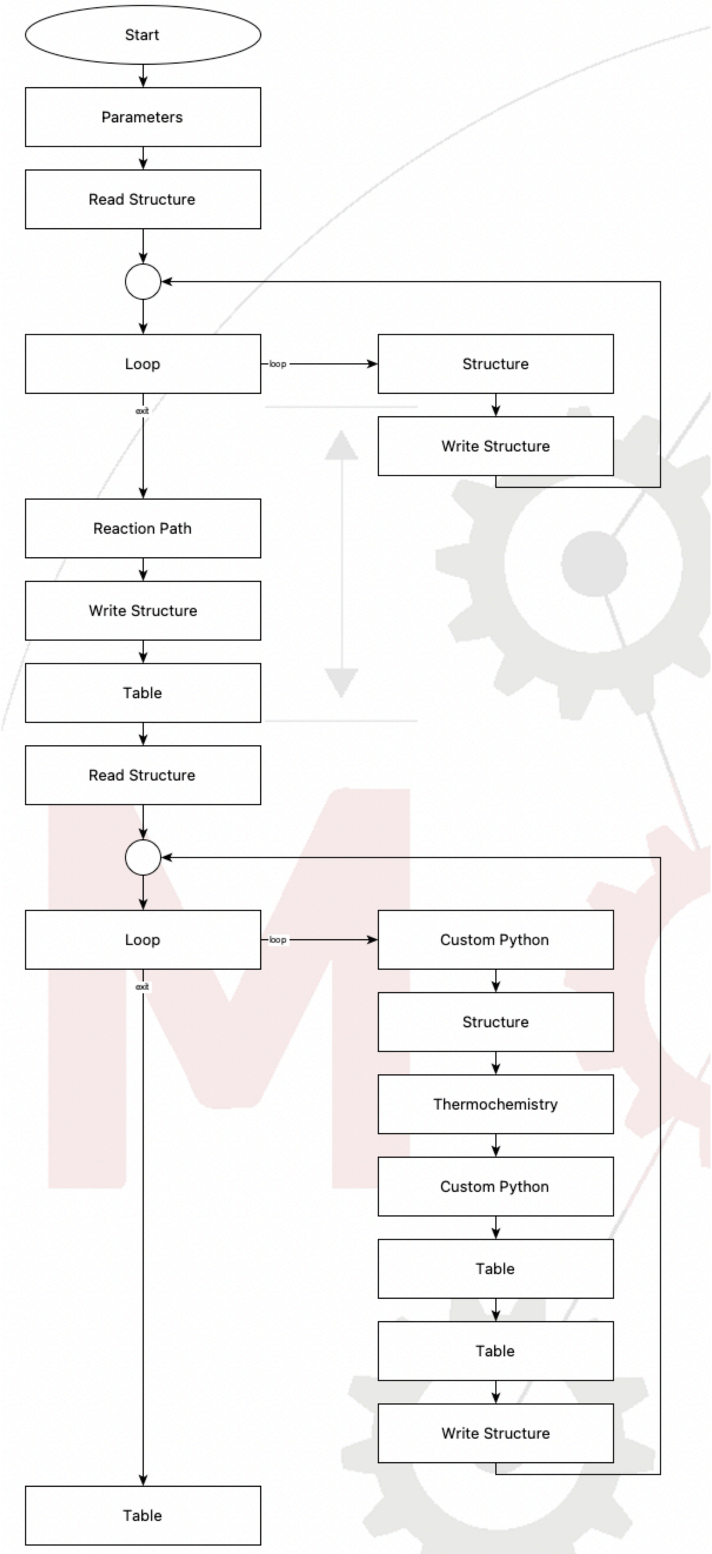

Figure 1: Flowchart for Reaction Thermochemistry

This flowchart uses loops to handle the initial structures as well as the final structures, `Table` steps to capture and tabulate the key results, and `Custom Python` steps to customize small parts of the flowchart. These steps will be covered in more detail below.

Initialization

The flowchart starts with a `Parameters` step that defines the value of variables that control parts of the flowchart. As mentioned, this makes the flowchart more general because the user can set the parameters when submitting the job, rather than edit the flowchart. Figure 2 shows the dialog for editing the control parameters.

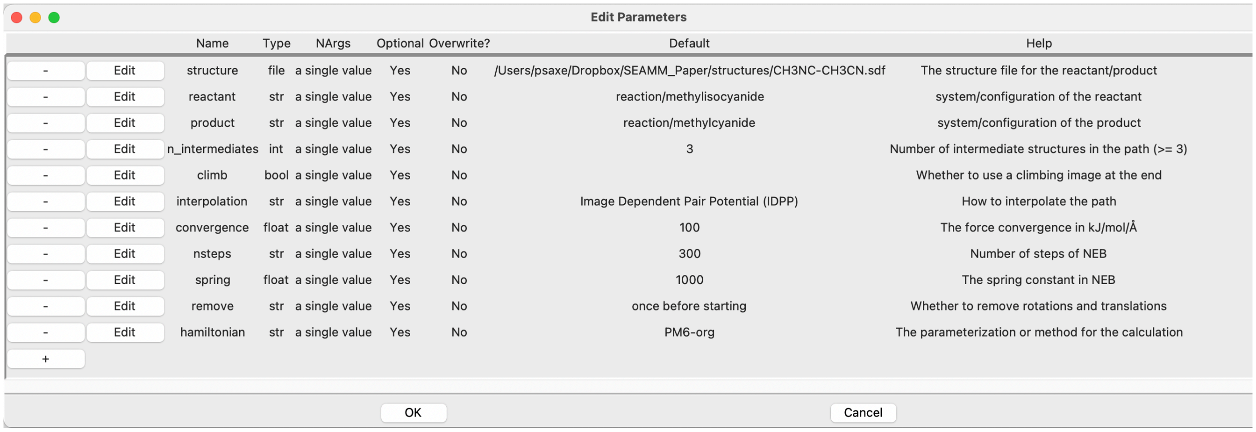

Edit Parameters

		Name	Type	NArgs	Optional	Overwrite?	Default	Help
-	Edit	structure	file	a single value	Yes	No	/Users/psaxe/Dropbox/SEAMM_Paper/structures/CH3NC-CH3CN.sdf	The structure file for the reactant/product
-	Edit	reactant	str	a single value	Yes	No	reaction/methylisocyanide	system/configuration of the reactant
-	Edit	product	str	a single value	Yes	No	reaction/methylcyanide	system/configuration of the product
-	Edit	n_intermediates	int	a single value	Yes	No	3	Number of intermediate structures in the path (>= 3)
-	Edit	climb	bool	a single value	Yes	No		Whether to use a climbing image at the end
-	Edit	interpolation	str	a single value	Yes	No	Image Dependent Pair Potential (IDPP)	How to interpolate the path
-	Edit	convergence	float	a single value	Yes	No	100	The force convergence in kJ/mol/Å
-	Edit	nsteps	str	a single value	Yes	No	300	Number of steps of NEB
-	Edit	spring	float	a single value	Yes	No	1000	The spring constant in NEB
-	Edit	remove	str	a single value	Yes	No	once before starting	Whether to remove rotations and translations
-	Edit	hamiltonian	str	a single value	Yes	No	PM6-org	The parameterization or method for the calculation
+								

OK Cancel

Figure 2: Control parameters for the reaction path flowchart

The first three parameters define the reactants and products for the reaction by giving the file containing the structures — in this case a structure-data format (SDF) file — and the names of the structures in that file that are the reactants and products. The assumption is that these structures have been prepared and placed in a file, which gives considerable flexibility in defining the reactant and product systems. Using these variables to define the reactants and products makes the flowchart general and applicable to many different reactions.

The next group of parameters control the nudged elastic band (NEB) method[1–3] used to find an approximate transition state. The NEB employs a number of structures between the

reactants and products connected from the reactants through the intermediate structures to the products with an elastic band. *n_intermediates* defines how many structures to use between the reactants and products, while *spring* gives the force constant for the elastic bands between adjacent structures. *interpolation* determines the initial guess for the intermediate structures, which can currently be linear interpolation or the image dependent pair potential (IDPP)[4] method. *climb* controls whether to use the standard NEB method or climbing image nudged elastic band (CI-NEB)[5] where the highest energy image is allowed to climb upwards towards the transition state, rather than remaining roughly in the middle between its two adjacent intermediate structures. *remove* controls whether to remove rigid rotations and translations and rotations between the various structures, and if so whether to do so just at the beginning of the calculation or at each step. Finally, *convergence* and *nsteps* give the convergence criterion for the calculation and a maximum number of steps before giving up if the calculation does not converge.

The last parameter, *hamiltonian*, specifies the parametrization for semiempirical methods, or the method for *ab initio* methods, or the forcefield for molecular mechanics. This allows the flowchart to be used for a variety of different approaches without editing. This parameter is used in the all the steps from the initial structure optimization through the reaction path to the final structure optimization and thermochemistry calculation.

Together, these parameters give considerable flexibility in defining the calculations in the flowchart, allowing the user to tune the calculation to their needs.

Optimization of the Reactant and Product Structures

After reading the structure file, the flowchart continues with a loop over the structures in the file, which are then optimized using the `Structure` step, which wraps the open source code `geomeTRIC`[6] to handle the structural optimization. As has been mentioned, the `Structure`, as well as the `Reaction Path` and `Thermochemistry` steps rely on a subflowchart to calculate the energy and forces of the structure, as shown in Figure 3, which uses DFTB+ for the

energy and forces.

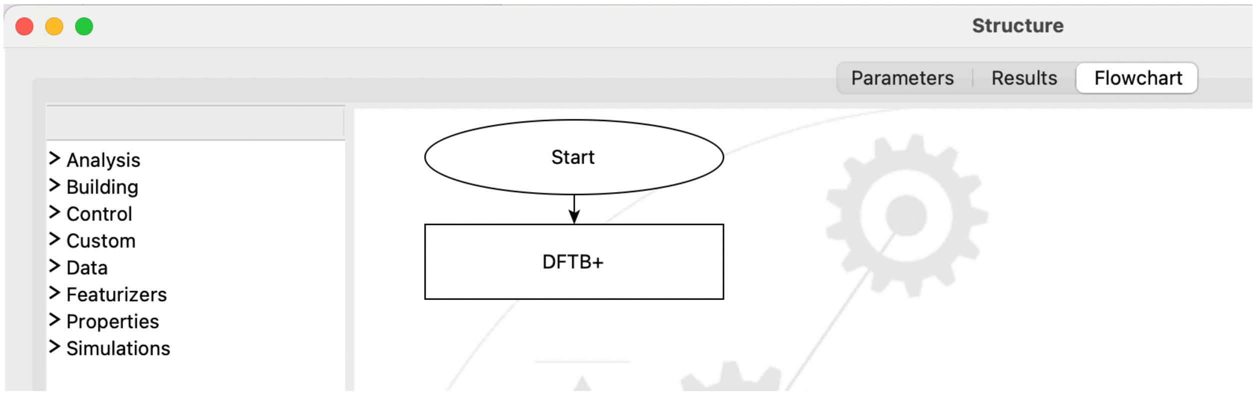

Figure 3: An example of the subflowchart common to the `Structure`, `Reaction Path`, `Thermochemistry`, and `Energy Scan` steps.

The flowchart to calculate the energy and forces using DFTB+ shown in Figure 4 is straightforward. Using any other simulation code to calculate the energy and forces is similarly simple, making it easy to switch between codes.

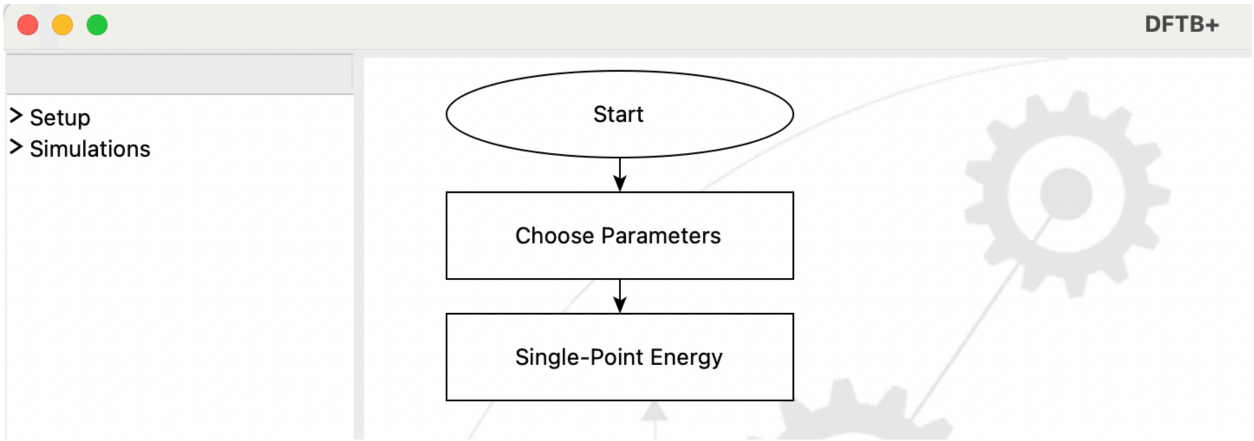

Figure 4: The flowchart to calculate the energy and forces using DFTB+.

The Reaction Path and Approximate Transition States

Once the structures for the reactants and products are optimized, the flowchart moves to the `Reaction Path` step, which uses the NEB and CI-NEB methods as implemented in Atomic Simulation Environment (ASE)[7] to find an approximate reaction path and transition state. The `Reaction Path` step uses an identical subflowchart to that shown above for

the `Structure` step. SEAMM supports copying and pasting flowcharts so the subflowchart can simply be copied from the `Structure` step. As noted above, several parameters controlling the NEB algorithm are defined by the initial `Parameters` step as variables, giving considerable flexibility in how to approach the NEB calculation.

As the steps in the flowchart run, the various structures that are generated are stored in the internal database in SEAMM for later use. In this flowchart the `Write Structure` step immediately after the `Reaction Path` step writes an SDF file with all the structures along the reaction path from the NEB calculation so that they can be viewed or used later. After creating a table with the `Table` step, which will be discussed a bit later, the flowchart proceeds to reading the structures for the stationary points that the `Reaction Path` step found and wrote to a specific SDF file. Note that while we have been talking as if there is a single transition state in the reaction path, i.e. the reaction is an elementary reaction, the NEB method is capable of handling more complex reactions with multiple transition states and intermediate structures between them. In such cases, all the stationary point structures would be output from the `Reaction Path` step and used in the subsequent steps of the flowchart.

Optimization of the Key Structures and Their Thermochemistry

The final `loop` step in the flow chart handles the optimization of the structures of the stationary points along the reaction path. If the reaction is an elementary reaction with just one transition state, there will be three structures: the reactants, the transition state, and the products. For more complicated reactions, there would be additional transition states as well as intermediate minima between them. In all cases, the loop optimizes each structure in the `Structure` step, calculating the thermochemical functions for each structure in the Thermochemistry step, which uses the identical subflowchart to the initial `Structure` step, which can be copied and pasted into this step. Internally the `Thermochemistry` step is using the `thermochemistry` module from ASE[7] to calculate the harmonic approximation to the

thermochemical functions of the vibrational frequencies.

There is a small issue with the optimizer that the `Custom Step` at the beginning of the loop solves. The optimizer in the `Structure` step needs to know whether it is targeting a minimum or a transition state. At the moment, the `Thermochemistry` step requires the symmetry number for the structure. In a future release, the `Thermochemistry` step will be enhanced to automatically determine the symmetry number, but for the moment it needs to be set manually or using a variable as shown in Figure 5.

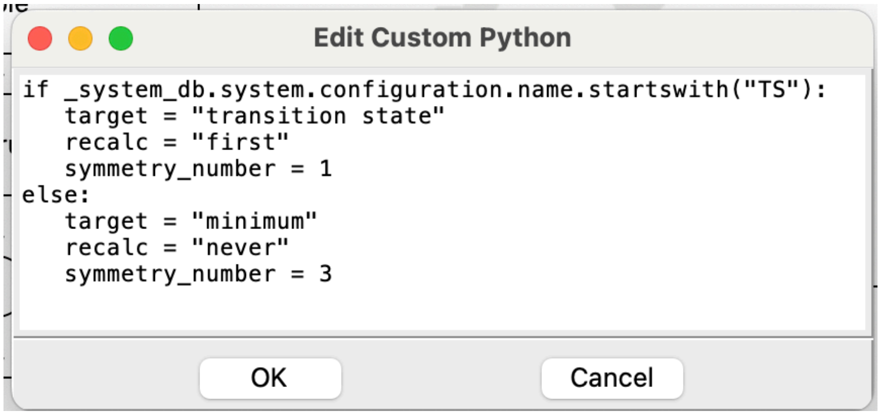

Figure 5: The Python code to set the required parameters for the `Structure` and `Thermochemistry` steps.

The somewhat cryptic first line is accessing the database in SEAMM (_system_db) and getting the name of the current *system* and *configuration*, which the `Reaction Step` ensures starts with “TS” if it is a transition state. The rest of the Python is self-explanatory. Unfortunately hard-wiring the symmetry number in this way makes the flowchart much less general, and is a temporary fix until the code can correctly determine the symmetry number from the structure.

We are almost done! The last part of the loop manipulates the energies that the `Structure` and `Thermochemistry` saved into variables, which can be accessed by any subsequent step, and then the two `Table` steps add a row to the table and store it as a comma separated values (CSV) file to capture the key results in a convenient form for the user. Figure 6 shows the `Custom` step that transforms the computed energies and enthalpies into

values relative to the reactants, and also captures the imaginary frequency of the transition states.

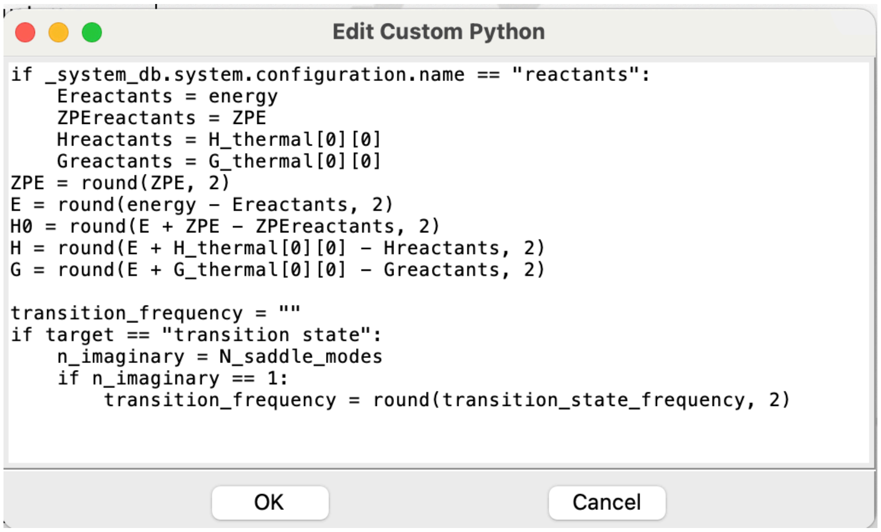

Figure 6: The Python code handle the computed energies and enthalpies.

This is quite straightforward but shows the value of being able to customize the flowchart with a little bit of Python code. In this case, we are able to transform the energies into relative energies, which the next step adds to the table, as shown in Figure 7.

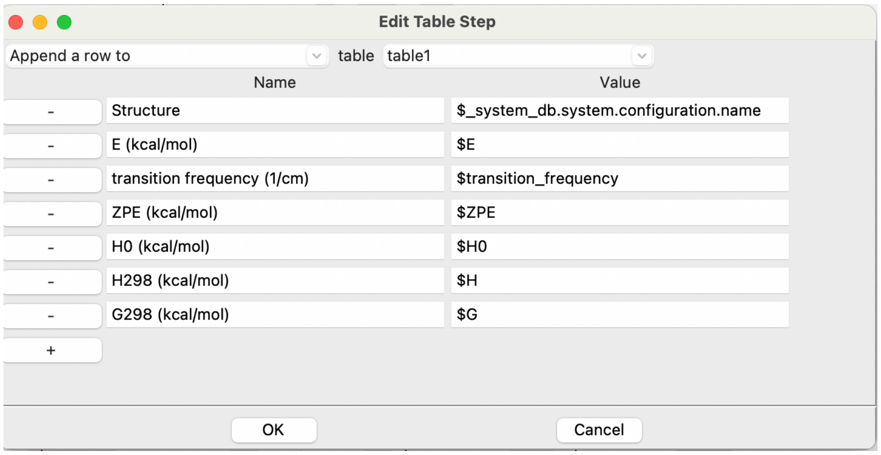

Figure 7: Adding a row of results to the table.

Note that the `Table` step is using the variables that were just defined in the `Custom` step shown in Figure 6. This illustrates how the Python in `Custom` steps can access variables set by other steps, and how variables can be used in any of the fields in dialogs if preceded by

a dollar sign ('$') to indicate that it is a variable rather than a literal value. This ability to define and use variables, and insert small fragments of custom code, greatly enhance the capabilities of SEAMM and its flowcharts.

Reaction path for the Rearrangement of Methylisocyanide to Isonitrile

This flowchart, with the appropriate subflowchart in the `Structure`, `Reaction Path`, and `Thermochemistry` steps, was used successfully with a number of *ab initio* and semiempirical Hartree-Fock and density functional theory (DFT) methods, as reported in the article and shown there in Table 2. These calculations ran smoothly and only had minor problems, mainly due to the different default accuracies of the various methods. Loose convergence criteria for the energy and gradients caused the optimization of the structure of the transition state to fail to converge. The tightening of the convergence criteria for the energy and gradients resolved these issues.

The situation when using several different version of the ReaxFF forcefield[8–10] and also different versions of the ANI parameterization of TorchANI[11,12] was quite different. The NEB algorithm failed to converge or produced an unreasonable reaction path. For example, in some cases the nitrogen-carbon triple bond of isonitrile broke, passing through a CH_3N + C structure, with the carbon atom then inserting between the methyl group and nitrogen to form isocyanide. In other cases, the methyl-nitrogen bond broke to yield CH_3 and CN which then recombined to form isonitrile. In another case, the structure exploded into separate atoms which then recombined to form the isonitrile. None of these paths is physically reasonable. We also noted that all of the forcefields and machine learning potentials predicted a bent structure for methylisocyanide, suggesting that the potentials had fundamental issues reproducing the isocyanide group.

Unfortunately it is not easy to determine whether the training set for the forcefields or

the machine learning potentials contained any isocyanides. Each of the forcefields used in this study were created by modifying existing forcefields to add new species and reactions. This required tracking the history of each forcefield and examining a number of papers and their supplementary information to understand the training set. As far as we can tell, none of the three ReaxFF forcefields examined contained an isocyanide in their training sets, but it is difficult to be certain.

The supplementary material associated with the ANI machine learning potentials contain the training sets used, which were subsets of the structures in available databases such as the GDB.[13] Although GDB was produced by an exhaustive generation of plausible structures containing up to, for example, 11 atoms of C, N, O, and F, and hence contains structures with isocyanide functional groups, the selection of a subset does not guarantee that the training set for the ANI potentials contains any isocyanides. The supplementary material, though complete, is only machine readable, so it requires writing a small program to access the data to determine the molecules in the training set and find that there are no isocyanides included. Having access to the training data makes it possible to definitively determine whether specific functional groups, such as isocyanide, were included in the training; however, the need to write a program to access the data is a considerable barrier.

Focusing on the methylisocyanide structure, we can see that the forcefields and ML potentials used cannot describe the structure well. As noted above, all methods predict a bent structure rather than the linear structure observed experimentally. In addition, some of the calculated vibrational frequencies, shown in Table 1, are nonphysical. In general the differences between the calculated and experimental frequencies strongly suggest that neither the ReaxFF forcefields nor the ANI ML potentials can reasonably describe methylisocyanide, let alone the transition state.

Table 1: The vibrational frequencies (cm^{-1}) calculated for methylisocyanide.

**Exptl.**[a]	**ReaxFF**[14]	**ReaxFF**[10]	**ReaxFF**[8]	**ANI-1x**[15]	**ANI-1cxx**[16]	**ANI-2x**[17]
263	66	11	68	431	222	258
263	288	488	394	981	850	835
945	485	1304	946	1394	1304	1145
1129	1293	1333	960	1532	1521	1172
1129	1294	1352	1367	1757	1698	1445
1419	1949	1832	1475	2019	2026	1918
1467	2075	1889	1475	2031	2045	1990
1467	2075	1894	2942	2155	2109	2921
2166	3309	2074	3104	3241	3233	4266
2966	3388	3253	3105	4258	4221	4459
3014	3388	3254	3237	4354	4243	4581
3014	6952	3320	9016	4391	4280	4980

[a] The experimental fundamental frequencies as quoted in Ref. 14.

Components of SEAMM

The framework of the SEAMM environment is composed of a number of different modules, as outlined in Table 2.

Table 2: The components of the SEAMM environment, including the current version.

	Component	Version	Description
1	molsystem	2025.5.19	The molecular/crystal data model
2	seamm	2025.5.27	The main part of SEAMM
3	seamm-ase	2025.2.7	Connector between SEAMM and ASE
4	seamm-dashboard	2024.6.2	The Web Dashboard
5	seamm-datastore	2024.6.2	Manages the data in the datastore for the Dashboard
6	seamm-exec	2025.6.23.2	Classes to execute background codes
7	seamm-ff-util	2025.5.26	Utility routines for handling forcefields
8	seamm-geometric	2025.5.7	Connector between geomeTRIC and SEAMM
9	seamm-installer	2025.6.21	The installer/updater for SEAMM
10	seamm-jobserver	2024.3.12	The JobServer for the SEAMM environment
11	seamm-util	2024.8.22	Utility methods
12	seamm-widgets	2024.10.10	Specialized tkinter widgets for SEAMM

The functionality of SEAMM is provided by plug-ins. Those currently available are listed in Table 3.

Table 3: The current plug-ins for SEAMM, including the current version

	Component	Version	Description
1	control-parameters-step	2025.5.7	for defining command-line parameters for a flowchart.
2	crystal-builder-step	2022.7.31	for creating crystals from prototypes, including Strukturbericht designations.
3	custom-step	2023.12.12	for custom Python scripts in a flowchart
4	dftbplus-step	2025.3.7	to setup, run and analyze calculations with DFTB+, a fast quantum mechanical simulation code.
5	diffusivity-step	2024.7.30.1	for calculating diffusivity
6	energy-scan-step	2025.5.7	for calculating energy profiles along coordinates
7	fhi-aims-step	2024.10.31	to setup, run and analyze calculations with FHI-aims
8	forcefield-step	2025.6.23	for setting up a forcefield or EAM potentials for subsequent simulations.

9	from-smiles-step	2025.5.14	for creating structures from SMILES, InChI, InChIKey, or name.
10	gaussian-step	2025.1.31	to setup, run and analyze quantum chemical calculations with Gaussian
11	geometry-analysis-step	2025.3.4	for analysis of the geometry of (small) molecules
12	lammps-step	2025.6.23	to setup, run and analyze calculations with LAMMPS, a forcefield-based molecular dynamics (MD) code.
13	loop-step	2025.5.15	provides loops in flowcharts
14	mopac-step	2025.5.7.1	to setup, run and analyze semiempirical calculations with MOPAC
15	packmol-step	2025.6.20	for building periodic boxes of fluid using Packmol
16	psi4-step	2025.3.9	to setup, run and analyze quantum chemistry calculations using Psi4
17	qcarchive-step	2023.3.30	for connecting with QCArchive
18	quickmin-step	2025.2.24	for simple, quick minimization using a forcefield
19	rdkit-step	2023.2.22	for RDKit descriptors/features
20	reaction-path-step	2024.12.14	for finding transition states and reaction paths
21	read-structure-step	2025.5.14	to read and write structures in many file formats common in computational chemistry
22	set-cell-step	2021.10.14	for setting the periodic (unit) cell
23	strain-step	2022.11.7	for straining periodic systems
24	structure-step	2025.5.14	for optimizing structures based on energy
25	subflowchart-step	2025.3.4	for subflowcharts in flowcharts
26	supercell-step	2023.11.5	for building supercells of periodic systems
27	table-step	2025.6.1	for data tables in a flowchart.
28	thermal-conductivity-step	2024.6.3	for calculating thermal conductivity
29	thermochemistry-step	2025.1.31	for calculating thermochemical functions
30	torchani-step	2025.3.10	for the TorchANI ML models

Understanding versioning in SEAMM

There is no single version number for SEAMM because SEAMM is composed of many different components that work together, as was outlined in the previous section. However, SEAMM provides several ways to track the versions of the components as well as to recreate

the installation if needed. This section will describe the available information.

First, note that the job.out file contains the version of the plug-ins used. In Example 1 in the paper, the summary of the job at the beginning of job.out is show in Figure 8.

```
Tuesday 2025.06.24 14:40:57
Running in directory '/Users/psaxe/SEAMM/Jobs/projects/Paper/Job_000235'

Description of the flowchart
----------------------------
Step 0: Start  2025.5.27
Step 1: Forcefield  2025.5.26.1
    Read the forcefield file 'oplsaa.frc' and use the default forcefield.

Step 2: Packmol  2025.3.9
    Will create a cubic periodic cell containing the following molecules:

        +-------------+-------------+---------+
        |  Component  | Structure   |   Ratio |
        |-------------+-------------+---------|
        |    fluid    | CCO         |       1 |
        +-------------+-------------+---------+

    The dimensions of the region will be calculated from the density 0.7 g/ml.
    The number of molecules of the fluid will be obtained by rounding 2000 atoms
    to give a whole number of molecules with the requested ratios.

Step 3: LAMMPS  2025.5.27

    Step 3.1: Initialization
        Initialize the calculation with a cutoff of 10.0 Å, shifting the nonbond
        energies to 0 at the cutoff. If the system is periodic the best k-space
        acceleration method for the  molecular system will be chosen. The accuracy
        goal is 1.00e-05.

    Step 3.2: Velocities
        Set the velocities to give a temperature 298.15 K by using a random
        distribution. LAMMPS will remove any translational and, for molecular
        systems, rotational momentum (default). The random number generator will be
        initialized randomly.

    Step 3.3: NPT dynamics
        100.0 ps of canonical (NPT) dynamics at 298.15 K using a timestep of normal
        fs . The temperature will be controlled using a Nose-Hoover thermostat. The
        thermostat will use a drag factor of 0.0. The pressure will be controlled
        using a Nose-Hoover barostat.
```

Figure 8: The summary output from the Large-scale Atomic/Molecular Massively Parallel Simulator (LAMMPS) step.

Each step gives the plug-in used (without the -step extension of the package) and its

version, e.g. **Step 3: LAMMPS 2025.5.27**. The substeps within LAMMPS do not have a version numbers because they are parts of the LAMMPS step. The version of the Start step is the version of the seamm module; however, the core of SEAMM is composed of several components, as shown in Table 2 above. Currently the versions of the other core components are not reflected in the output.

Every time the installer installs or updates parts of SEAMM, it creates 3 time-stamped files in /SEAMM/environments that describe the release at that timepoint in various levels of detail. Listing the files in /SEAMM/environments shows a list of files like those in Figure 9

```
(seamm-dev) psaxe@PaulsPersonal environments % ls -ltr
ls -ltr
total 832
-rw-r--r--  1 psaxe  staff   1861 Jun  6 14:43 2025-06-06T14:43:19_install.yml
-rw-r--r--  1 psaxe  staff   9332 Jun  6 14:44 2025-06-06T14:43:19_environment.yml
-rw-r--r--  1 psaxe  staff  18994 Jun  6 14:44 2025-06-06T14:43:19_environment.txt
-rw-r--r--  1 psaxe  staff   1068 Jun 25 10:44 2025-06-25T10:44:00_update.yml
-rw-r--r--  1 psaxe  staff   9317 Jun 25 10:44 2025-06-25T10:44:00_environment.yml
-rw-r--r--  1 psaxe  staff  18994 Jun 25 10:44 2025-06-25T10:44:00_environment.txt
(seamm-dev) psaxe@PaulsPersonal environments %
```

Figure 9: Files in /SEAMM/environments.

There are three files for each timestamp. The suffix of the first file indicates whether the installer was installing, updating, etc. An example of this type of file for the initial installation is shown in Liste 1 The remaining pair of files are the state of the environment after the installer completed the task. The .yml file is a list of all the files in the release, including the python version and all the libraries used, pinned to their exact version. An example is shown in Listing 2. The last file, the .txt file is exactly the Conda packages installed on the current machine, and can be used to precisely recreate the environment; however, it is specific to the architecture of the current machine, so might not work on other hardware. An example is shown in Listing 3. The .yml file is more general and should work on most hardware.

We can see from the file list that SEAMM was installed on 6 June 2025 and updated on

25 June, and we can, if needed, recreate the environment at any of those points.

Listing 1: The machine-dependent environment file
2025-06-06T14:43:19_install.yml

```
name: seamm
channels:
  - conda-forge
  - defaults
dependencies:
  - pip
  - python

  # Core packages
  - molsystem
  - seamm
  - seamm-dashboard
  - seamm-datastore
  - seamm-ff-util

  # MolSSI plug-ins

  # 3rd-party plug-ins

  # Dependencies that require special handling

  # molsystem: libsqlite version 3.49.1 is badly broken!
  - libsqlite!=3.49.1
  # molsystem: Currently ommitted from molsystem requirements...
  - pubchempy
  # seamm: pip cannot install, so insist on conda
  - psutil
  # seamm-dashboard: Dashboard fails with version 3, so...
  - connexion<3.0
  # seamm-dashboard: Later version caused problems logging in
  - flask-jwt-extended=4.5.3
  # seamm-dashboard: Later version caused problems logging in
  - pyjwt=2.9.0
  # qcarchive-step: qcportal requires apsw, which must be compiled if using pip.
  - qcportal

  # PyPi packages
  - pip:
    # Core packages
    - seamm-ase
    - seamm-exec
    - seamm-geometric
    - seamm-jobserver
```

```
# MolSSI plug-ins
- control-parameters-step
- crystal-builder-step
- custom-step
- dftbplus-step
- diffusivity-step
- energy-scan-step
- fhi-aims-step
- forcefield-step
- from-smiles-step
- gaussian-step
- geometry-analysis-step
- lammps-step
- loop-step
- mopac-step
- packmol-step
- psi4-step
- qcarchive-step
- quickmin-step
- rdkit-step
- reaction-path-step
- read-structure-step
- set-cell-step
- strain-step
- structure-step
- subflowchart-step
- supercell-step
- table-step
- thermal-conductivity-step
- thermochemistry-step
- torchani-step

# 3rd-party plug-ins

# Dependencies that require special handling

# seamm: conda-forge version is old and does not work
- pmw
```

Listing 2: The machine-independent environment file 2025-06-06T14:43:19_environment.yml


```
name: seamm
channels:
  - conda-forge
  - defaults
dependencies:
  - alembic=1.16.1=pyhd8ed1ab_0
  - amqp=5.2.0=pyhd8ed1ab_2
  - annotated-types=0.7.0=pyhd8ed1ab_1
  - attrs=25.3.0=pyh71513ae_0
  - backports-datetime-fromisoformat=2.0.3=py312h81bd7bf_0
  - bibtexparser=1.4.3=pyhd8ed1ab_0
  - billiard=4.2.1=py312h024a12e_0
  - blinker=1.9.0=pyhff2d567_0
  - boto3=1.38.31=pyhd8ed1ab_0
  - botocore=1.38.31=pyge310_1234567_0
  - brotli=1.1.0=h5505292_3
  - brotli-bin=1.1.0=h5505292_3
  - brotli-python=1.1.0=py312hd8f9ff3_3
  - bzip2=1.0.8=h99b78c6_7
  - ca-certificates=2025.4.26=hbd8a1cb_0
  - cairo=1.18.4=h6a3b0d2_0
  - celery=5.5.3=pyhe01879c_0
  - certifi=2025.4.26=pyhd8ed1ab_0
  - cffi=1.17.1=py312h0fad829_0
  - chardet=5.2.0=pyhd8ed1ab_3
  - charset-normalizer=3.4.2=pyhd8ed1ab_0
  - click=8.2.1=pyh707e725_0
  - click-didyoumean=0.3.1=pyhd8ed1ab_1
  - click-plugins=1.1.1=pyhd8ed1ab_1
  - click-repl=0.3.0=pyhd8ed1ab_0
  - clickclick=20.10.2=pyhd8ed1ab_1
  - colorama=0.4.6=pyhd8ed1ab_1
  - connexion=2.14.2=pyhd8ed1ab_1
  - contourpy=1.3.2=py312hb23fbb9_0
  - cpython=3.12.11=py312hd8ed1ab_0
  - cycler=0.12.1=pyhd8ed1ab_1
  - cyrus-sasl=2.1.27=h60b93bd_7
  - dnspython=2.7.0=pyhff2d567_1
  - dominate=2.9.1=pyhd8ed1ab_1
  - email-validator=2.2.0=pyhd8ed1ab_1
  - fasteners=0.19=pyhd8ed1ab_1
  - flask=2.2.5=pyhd8ed1ab_0
  - flask-authorize=0.2.6=pyhd8ed1ab_0
  - flask-bootstrap=3.3.7.1=py_0
```

```
- flask-compress=1.17=pyhd8ed1ab_1
- flask-cors=6.0.0=pyhe01879c_0
- flask-jwt-extended=4.5.3=pyhd8ed1ab_0
- flask-mail=0.10.0=pyhd8ed1ab_0
- flask-marshmallow=1.3.0=pyhd8ed1ab_0
- flask-moment=1.0.6=pyhd8ed1ab_1
- flask-sqlalchemy=3.0.3=pyhd8ed1ab_0
- flask-wtf=1.2.2=pyhd8ed1ab_1
- flexcache=0.3=pyhd8ed1ab_1
- flexparser=0.4=pyhd8ed1ab_1
- font-ttf-dejavu-sans-mono=2.37=hab24e00_0
- font-ttf-inconsolata=3.000=h77eed37_0
- font-ttf-source-code-pro=2.038=h77eed37_0
- font-ttf-ubuntu=0.83=h77eed37_3
- fontconfig=2.15.0=h1383a14_1
- fonts-conda-ecosystem=1=0
- fonts-conda-forge=1=0
- fonttools=4.58.2=py312h998013c_0
- freetype=2.13.3=hce30654_1
- freetype-py=2.3.0=pyhd8ed1ab_0
- future=1.0.0=pyhd8ed1ab_2
- gmp=6.3.0=h7bae524_2
- gmpy2=2.2.1=py312h524cf62_0
- greenlet=3.2.3=py312hd8f9ff3_0
- h2=4.2.0=pyhd8ed1ab_0
- hpack=4.1.0=pyhd8ed1ab_0
- humanize=4.12.3=pyhd8ed1ab_0
- hyperframe=6.1.0=pyhd8ed1ab_0
- icu=75.1=hfee45f7_0
- idna=3.10=pyhd8ed1ab_1
- importlib-metadata=8.7.0=pyhe01879c_1
- importlib-resources=6.5.2=pyhd8ed1ab_0
- importlib_resources=6.5.2=pyhd8ed1ab_0
- inflection=0.5.1=pyhd8ed1ab_1
- itsdangerous=2.2.0=pyhd8ed1ab_1
- jinja2=3.1.6=pyhd8ed1ab_0
- jmespath=1.0.1=pyhd8ed1ab_1
- jsonschema=4.24.0=pyhd8ed1ab_0
- jsonschema-specifications=2025.4.1=pyh29332c3_0
- kiwisolver=1.4.8=py312h2c4a281_0
- kombu=5.5.4=pyha770c72_0
- krb5=1.21.3=h237132a_0
- lcms2=2.17=h7eeda09_0
- lerc=4.0.0=hd64df32_1
- libblas=3.9.0=31_h10e41b3_openblas
- libboost=1.86.0=hc9fb7c5_3
- libboost-python=1.86.0=py312h72cd453_3
```

```
- libbrotlicommon=1.1.0=h5505292_3
- libbrotlidec=1.1.0=h5505292_3
- libbrotlienc=1.1.0=h5505292_3
- libcblas=3.9.0=31_hb3479ef_openblas
- libcxx=20.1.6=ha82da77_0
- libdeflate=1.24=h5773f1b_0
- libedit=3.1.20250104=pl5321hafb1f1b_0
- libexpat=2.7.0=h286801f_0
- libffi=3.4.6=h1da3d7d_1
- libfreetype=2.13.3=hce30654_1
- libfreetype6=2.13.3=h1d14073_1
- libgfortran=14.2.0=heb5dd2a_105
- libgfortran5=14.2.0=h2c44a93_105
- libglib=2.84.2=hbec27ea_0
- libiconv=1.18=hfe07756_1
- libintl=0.24.1=h493aca8_0
- libjpeg-turbo=3.1.0=h5505292_0
- liblapack=3.9.0=31_hc9a63f6_openblas
- liblzma=5.8.1=h39f12f2_2
- libntlm=1.8=h5505292_0
- libopenblas=0.3.29=openmp_hf332438_0
- libpng=1.6.47=h3783ad8_0
- libpq=17.5=h6896619_0
- librdkit=2025.03.3=hab0a279_0
- libsqlite=3.50.1=h3f77e49_0
- libtiff=4.7.0=h2f21f7c_5
- libwebp-base=1.5.0=h2471fea_0
- libxcb=1.17.0=hdb1d25a_0
- libxml2=2.13.8=h52572c6_0
- libzlib=1.3.1=h8359307_2
- llvm-openmp=20.1.6=hdb05f8b_0
- mako=1.3.10=pyhd8ed1ab_0
- markupsafe=3.0.2=py312h998013c_1
- marshmallow=4.0.0=pyhd8ed1ab_0
- marshmallow-sqlalchemy=1.4.2=pyhd8ed1ab_0
- matplotlib-base=3.10.3=py312hdbc7e53_0
- molsystem=2025.5.19=pyhd8ed1ab_0
- mpc=1.3.1=h8f1351a_1
- mpfr=4.2.1=hb693164_3
- mpmath=1.3.0=pyhd8ed1ab_1
- msgpack-python=1.1.0=py312h6142ec9_0
- munkres=1.1.4=pyh9f0ad1d_0
- ncurses=6.5=h5e97a16_3
- numpy=2.2.6=py312h7c1f314_0
- openbabel=3.1.1=py312h2eeb2e4_9
- openjpeg=2.5.3=h8a3d83b_0
- openldap=2.6.10=hbe55e7a_0
```

```
- openssl=3.5.0=h81ee809_1
- packaging=25.0=pyh29332c3_1
- pandas=2.2.3=py312hcd31e36_1
- pathvalidate=3.2.3=pyhd8ed1ab_0
- patsy=1.0.1=pyhd8ed1ab_1
- pbr=6.1.1=pyhd8ed1ab_1
- pcre2=10.45=ha881caa_0
- pillow=11.2.1=py312h50aef2c_0
- pint=0.24.4=pyhd8ed1ab_1
- pip=25.1.1=pyh8b19718_0
- pixman=0.46.0=h2f9eb0b_0
- pkgutil-resolve-name=1.3.10=pyhd8ed1ab_2
- platformdirs=4.3.8=pyhe01879c_0
- ply=3.11=pyhd8ed1ab_3
- prompt-toolkit=3.0.51=pyha770c72_0
- prompt_toolkit=3.0.51=hd8ed1ab_0
- psutil=7.0.0=py312hea69d52_0
- pthread-stubs=0.4=hd74edd7_1002
- pubchempy=1.0.4=pyhd8ed1ab_1
- py-cpuinfo=9.0.0=pyhd8ed1ab_1
- pycairo=1.28.0=py312h0437987_0
- pycifrw=4.4.6=py312h024a12e_2
- pycparser=2.22=pyh29332c3_1
- pydantic=2.11.4=pyh3cfb1c2_0
- pydantic-core=2.33.2=py312hd3c0895_0
- pyjwt=2.9.0=pyhd8ed1ab_1
- pyobjc-core=11.0=py312hb9d441b_0
- pyobjc-framework-cocoa=11.0=py312hb9d441b_0
- pyparsing=3.2.3=pyhd8ed1ab_1
- pyperclip=1.9.0=pyh534df25_3
- pysocks=1.7.1=pyha55dd90_7
- python=3.12.11=hc22306f_0_cpython
- python-dateutil=2.9.0.post0=pyhff2d567_1
- python-tzdata=2025.2=pyhd8ed1ab_0
- python_abi=3.12=7_cp312
- pytz=2024.1=pyhd8ed1ab_0
- pyuca=1.2=pyhd8ed1ab_2
- pyyaml=6.0.2=py312h998013c_2
- qcelemental=0.29.0=pyhd8ed1ab_1
- qcportal=0.54.1=pyhd8ed1ab_0
- qhull=2020.2=h420ef59_5
- rdkit=2025.03.3=py312had33d2d_0
- readline=8.2=h1d1bf99_2
- reference-handler=0.9.1=pyhd8ed1ab_0
- referencing=0.36.2=pyh29332c3_0
- reportlab=4.4.1=py312hea69d52_0
- requests=2.32.3=pyhd8ed1ab_1
```

```
- requests-toolbelt=1.0.0=pyhd8ed1ab_1
- rlpycairo=0.2.0=pyhd8ed1ab_0
- rpds-py=0.25.1=py312hd3c0895_0
- s3transfer=0.13.0=pyhd8ed1ab_0
- scipy=1.15.2=py312h99a188d_0
- seamm=2025.5.27=pyhd8ed1ab_0
- seamm-dashboard=2024.6.2=pyhd8ed1ab_0
- seamm-dashboard-client=2024.6.27=pyhff2d567_0
- seamm-datastore=2024.6.2=pyhd8ed1ab_0
- seamm-ff-util=2025.5.26=pyhd8ed1ab_0
- seamm-util=2024.8.22=pyhd8ed1ab_1
- seamm-widgets=2024.10.10=pyhd8ed1ab_0
- seekpath=2.1.0=pyhd8ed1ab_1
- semver=3.0.4=pyhd8ed1ab_0
- setuptools=80.9.0=pyhff2d567_0
- six=1.17.0=pyhd8ed1ab_0
- sniffio=1.3.1=pyhd8ed1ab_1
- spglib=2.6.0=py312h3daaf81_0
- sqlalchemy=1.4.54=py312hea69d52_0
- statsmodels=0.14.4=py312h755e627_0
- stevedore=5.4.1=pyhd8ed1ab_0
- swagger-ui-bundle=1.1.0=pyhd8ed1ab_1
- sympy=1.14.0=pyh2585a3b_105
- tabulate=0.9.0=pyhd8ed1ab_2
- tk=8.6.13=h892fb3f_2
- tomli=2.2.1=pyhd8ed1ab_1
- tqdm=4.67.1=pyhd8ed1ab_1
- typing-extensions=4.14.0=h32cad80_0
- typing-inspection=0.4.1=pyhd8ed1ab_0
- typing_extensions=4.14.0=pyhe01879c_0
- tzdata=2025b=h78e105d_0
- unicodedata2=16.0.0=py312hea69d52_0
- urllib3=2.4.0=pyhd8ed1ab_0
- vine=5.1.0=pyhd8ed1ab_1
- visitor=0.1.3=pyhd8ed1ab_2
- waitress=3.0.2=pyhd8ed1ab_1
- wcwidth=0.2.13=pyhd8ed1ab_1
- werkzeug=2.2.3=pyhd8ed1ab_0
- wheel=0.45.1=pyhd8ed1ab_1
- wtforms=3.2.1=pyhd8ed1ab_1
- xorg-libxau=1.0.12=h5505292_0
- xorg-libxdmcp=1.1.5=hd74edd7_0
- yaml=0.2.5=h3422bc3_2
- zipp=3.22.0=pyhd8ed1ab_0
- zstandard=0.23.0=py312hea69d52_2
- zstd=1.5.7=h6491c7d_2
- pip:
```

```
- ase==3.25.0
- cclib==1.8.1
- cms-plots==2025.5.7
- control-parameters-step==2025.5.7
- crystal-builder-step==2022.7.31
- custom-step==2023.12.12
- dftbplus-step==2025.3.7
- diffusivity-step==2024.7.30.1
- docker==7.1.0
- energy-scan-step==2025.5.7
- et-xmlfile==2.0.0
- fhi-aims-step==2024.10.31
- forcefield-step==2025.5.26.1
- from-smiles-step==2025.5.14
- gaussian-step==2025.1.31
- geometric==1.1
- geometry-analysis-step==2025.3.4
- hsd==0.1
- kim-query==4.0.0
- lammps-step==2025.5.27
- loop-step==2025.5.15
- mopac-step==2025.5.7.1
- networkx==3.5
- numexpr==2.10.2
- openpyxl==3.1.5
- packmol-step==2025.3.9
- periodictable==2.0.2
- pmw==2.1.1
- psi4-step==2025.3.9
- pymbar==4.0.3
- qcarchive-step==2023.3.30
- quickmin-step==2025.2.24
- rdkit-step==2023.2.22
- reaction-path-step==2024.12.14
- read-structure-step==2025.5.14
- seamm-ase==2025.2.7
- seamm-exec==2025.1.3
- seamm-geometric==2025.5.7
- seamm-installer==2025.4.30+0.gb8adddf.dirty
- seamm-jobserver==2024.3.12
- set-cell-step==2021.10.14
- strain-step==2022.11.7
- structure-step==2025.5.14
- subflowchart-step==2025.3.4
- supercell-step==2023.11.5
- table-step==2025.6.1
- thermal-conductivity-step==2024.6.3
```

```
      - thermochemistry-step==2025.1.31
      - torchani-step==2025.3.10
prefix: /Users/psaxe/miniconda3/envs/seamm
```

Listing 3: The machine-dependent environment file
2025-06-06T14:43:19_environment.txt

```
# This file may be used to create an environment using:
# $ conda create --name <env> --file <this file>
# platform: osx-arm64
# created-by: conda 24.9.2
@EXPLICIT
https://conda.anaconda.org/conda-forge/osx-arm64/bzip2-1.0.8-h99b78c6_7.conda
https://conda.anaconda.org/conda-forge/noarch/ca-certificates-2025.4.26-hbd8a1cb_0.conda
https://conda.anaconda.org/conda-forge/noarch/font-ttf-dejavu-sans-mono-2.37-hab24e00_0.tar.bz2
https://conda.anaconda.org/conda-forge/noarch/font-ttf-inconsolata-3.000-h77eed37_0.tar.bz2
https://conda.anaconda.org/conda-forge/noarch/font-ttf-source-code-pro-2.038-h77eed37_0.tar.bz2
https://conda.anaconda.org/conda-forge/noarch/font-ttf-ubuntu-0.83-h77eed37_3.conda
https://conda.anaconda.org/conda-forge/osx-arm64/icu-75.1-hfee45f7_0.conda
https://conda.anaconda.org/conda-forge/osx-arm64/libbrotlicommon-1.1.0-h5505292_3.conda
https://conda.anaconda.org/conda-forge/osx-arm64/libcxx-20.1.6-ha82da77_0.conda
https://conda.anaconda.org/conda-forge/osx-arm64/libdeflate-1.24-h5773f1b_0.conda
https://conda.anaconda.org/conda-forge/osx-arm64/libexpat-2.7.0-h286801f_0.conda
https://conda.anaconda.org/conda-forge/osx-arm64/libffi-3.4.6-h1da3d7d_1.conda
https://conda.anaconda.org/conda-forge/osx-arm64/libiconv-1.18-hfe07756_1.conda
https://conda.anaconda.org/conda-forge/osx-arm64/libjpeg-turbo-3.1.0-h5505292_0.conda
https://conda.anaconda.org/conda-forge/osx-arm64/liblzma-5.8.1-h39f12f2_2.conda
https://conda.anaconda.org/conda-forge/osx-arm64/libntlm-1.8-h5505292_0.conda
https://conda.anaconda.org/conda-forge/osx-arm64/libwebp-base-1.5.0-h2471fea_0.conda
https://conda.anaconda.org/conda-forge/osx-arm64/libzlib-1.3.1-h8359307_2.conda
https://conda.anaconda.org/conda-forge/osx-arm64/llvm-openmp-20.1.6-hdb05f8b_0.conda
https://conda.anaconda.org/conda-forge/osx-arm64/ncurses-6.5-h5e97a16_3.conda
https://conda.anaconda.org/conda-forge/osx-arm64/pthread-stubs-0.4-hd74edd7_1002.conda
https://conda.anaconda.org/conda-forge/noarch/python_abi-3.12-7_cp312.conda
https://conda.anaconda.org/conda-forge/noarch/tzdata-2025b-h78e105d_0.conda
https://conda.anaconda.org/conda-forge/osx-arm64/xorg-libxau-1.0.12-h5505292_0.conda
https://conda.anaconda.org/conda-forge/osx-arm64/xorg-libxdmcp-1.1.5-hd74edd7_0.conda
https://conda.anaconda.org/conda-forge/osx-arm64/yaml-0.2.5-h3422bc3_2.tar.bz2
https://conda.anaconda.org/conda-forge/noarch/fonts-conda-forge-1-0.tar.bz2
https://conda.anaconda.org/conda-forge/osx-arm64/gmp-6.3.0-h7bae524_2.conda
https://conda.anaconda.org/conda-forge/osx-arm64/lerc-4.0.0-hd64df32_1.conda
https://conda.anaconda.org/conda-forge/osx-arm64/libbrotlidec-1.1.0-h5505292_3.conda
https://conda.anaconda.org/conda-forge/osx-arm64/libbrotlienc-1.1.0-h5505292_3.conda
https://conda.anaconda.org/conda-forge/osx-arm64/libedit-3.1.20250104-pl5321hafb1f1b_0.conda
https://conda.anaconda.org/conda-forge/osx-arm64/libgfortran5-14.2.0-h2c44a93_105.conda
https://conda.anaconda.org/conda-forge/osx-arm64/libintl-0.24.1-h493aca8_0.conda
https://conda.anaconda.org/conda-forge/osx-arm64/libpng-1.6.47-h3783ad8_0.conda
https://conda.anaconda.org/conda-forge/osx-arm64/libsqlite-3.50.1-h3f77e49_0.conda
https://conda.anaconda.org/conda-forge/osx-arm64/libxcb-1.17.0-hdb1d25a_0.conda
https://conda.anaconda.org/conda-forge/osx-arm64/libxml2-2.13.8-h52572c6_0.conda
https://conda.anaconda.org/conda-forge/osx-arm64/openssl-3.5.0-h81ee809_1.conda
```

```
https://conda.anaconda.org/conda-forge/osx-arm64/pcre2-10.45-ha881caa_0.conda
https://conda.anaconda.org/conda-forge/osx-arm64/pixman-0.46.0-h2f9eb0b_0.conda
https://conda.anaconda.org/conda-forge/osx-arm64/qhull-2020.2-h420ef59_5.conda
https://conda.anaconda.org/conda-forge/osx-arm64/readline-8.2-h1d1bf99_2.conda
https://conda.anaconda.org/conda-forge/osx-arm64/tk-8.6.13-h892fb3f_2.conda
https://conda.anaconda.org/conda-forge/osx-arm64/zstd-1.5.7-h6491c7d_2.conda
https://conda.anaconda.org/conda-forge/osx-arm64/brotli-bin-1.1.0-h5505292_3.conda
https://conda.anaconda.org/conda-forge/noarch/fonts-conda-ecosystem-1-0.tar.bz2
https://conda.anaconda.org/conda-forge/osx-arm64/krb5-1.21.3-h237132a_0.conda
https://conda.anaconda.org/conda-forge/osx-arm64/libboost-1.86.0-hc9fb7c5_3.conda
https://conda.anaconda.org/conda-forge/osx-arm64/libfreetype6-2.13.3-h1d14073_1.conda
https://conda.anaconda.org/conda-forge/osx-arm64/libgfortran-14.2.0-heb5dd2a_105.conda
https://conda.anaconda.org/conda-forge/osx-arm64/libglib-2.84.2-hbec27ea_0.conda
https://conda.anaconda.org/conda-forge/osx-arm64/libtiff-4.7.0-h2f21f7c_5.conda
https://conda.anaconda.org/conda-forge/osx-arm64/mpfr-4.2.1-hb693164_3.conda
https://conda.anaconda.org/conda-forge/osx-arm64/python-3.12.11-hc22306f_0_cpython.conda
https://conda.anaconda.org/conda-forge/noarch/attrs-25.3.0-pyh71513ae_0.conda
https://conda.anaconda.org/conda-forge/osx-arm64/backports-datetime-fromisoformat-2.0.3-py312h81
https://conda.anaconda.org/conda-forge/osx-arm64/billiard-4.2.1-py312h024a12e_0.conda
https://conda.anaconda.org/conda-forge/noarch/blinker-1.9.0-pyhff2d567_0.conda
https://conda.anaconda.org/conda-forge/osx-arm64/brotli-1.1.0-h5505292_3.conda
https://conda.anaconda.org/conda-forge/osx-arm64/brotli-python-1.1.0-py312hd8f9ff3_3.conda
https://conda.anaconda.org/conda-forge/noarch/certifi-2025.4.26-pyhd8ed1ab_0.conda
https://conda.anaconda.org/conda-forge/noarch/chardet-5.2.0-pyhd8ed1ab_3.conda
https://conda.anaconda.org/conda-forge/noarch/charset-normalizer-3.4.2-pyhd8ed1ab_0.conda
https://conda.anaconda.org/conda-forge/noarch/click-8.2.1-pyh707e725_0.conda
https://conda.anaconda.org/conda-forge/noarch/colorama-0.4.6-pyhd8ed1ab_1.conda
https://conda.anaconda.org/conda-forge/noarch/cpython-3.12.11-py312hd8ed1ab_0.conda
https://conda.anaconda.org/conda-forge/noarch/cycler-0.12.1-pyhd8ed1ab_1.conda
https://conda.anaconda.org/conda-forge/osx-arm64/cyrus-sasl-2.1.27-h60b93bd_7.conda
https://conda.anaconda.org/conda-forge/noarch/dominate-2.9.1-pyhd8ed1ab_1.conda
https://conda.anaconda.org/conda-forge/noarch/fasteners-0.19-pyhd8ed1ab_1.conda
https://conda.anaconda.org/conda-forge/noarch/future-1.0.0-pyhd8ed1ab_2.conda
https://conda.anaconda.org/conda-forge/osx-arm64/greenlet-3.2.3-py312hd8f9ff3_0.conda
https://conda.anaconda.org/conda-forge/noarch/hpack-4.1.0-pyhd8ed1ab_0.conda
https://conda.anaconda.org/conda-forge/noarch/humanize-4.12.3-pyhd8ed1ab_0.conda
https://conda.anaconda.org/conda-forge/noarch/hyperframe-6.1.0-pyhd8ed1ab_0.conda
https://conda.anaconda.org/conda-forge/noarch/idna-3.10-pyhd8ed1ab_1.conda
https://conda.anaconda.org/conda-forge/noarch/inflection-0.5.1-pyhd8ed1ab_1.conda
https://conda.anaconda.org/conda-forge/noarch/itsdangerous-2.2.0-pyhd8ed1ab_1.conda
https://conda.anaconda.org/conda-forge/noarch/jmespath-1.0.1-pyhd8ed1ab_1.conda
https://conda.anaconda.org/conda-forge/osx-arm64/kiwisolver-1.4.8-py312h2c4a281_0.conda
https://conda.anaconda.org/conda-forge/osx-arm64/lcms2-2.17-h7eeda09_0.conda
https://conda.anaconda.org/conda-forge/osx-arm64/libfreetype-2.13.3-hce30654_1.conda
https://conda.anaconda.org/conda-forge/osx-arm64/libopenblas-0.3.29-openmp_hf332438_0.conda
https://conda.anaconda.org/conda-forge/osx-arm64/markupsafe-3.0.2-py312h998013c_1.conda
https://conda.anaconda.org/conda-forge/osx-arm64/mpc-1.3.1-h8f1351a_1.conda
```

https://conda.anaconda.org/conda-forge/noarch/mpmath-1.3.0-pyhd8ed1ab_1.conda
https://conda.anaconda.org/conda-forge/osx-arm64/msgpack-python-1.1.0-py312h6142ec9_0.conda
https://conda.anaconda.org/conda-forge/noarch/munkres-1.1.4-pyh9f0ad1d_0.tar.bz2
https://conda.anaconda.org/conda-forge/osx-arm64/openjpeg-2.5.3-h8a3d83b_0.conda
https://conda.anaconda.org/conda-forge/noarch/packaging-25.0-pyh29332c3_1.conda
https://conda.anaconda.org/conda-forge/noarch/pathvalidate-3.2.3-pyhd8ed1ab_0.conda
https://conda.anaconda.org/conda-forge/noarch/pkgutil-resolve-name-1.3.10-pyhd8ed1ab_2.conda
https://conda.anaconda.org/conda-forge/noarch/platformdirs-4.3.8-pyhe01879c_0.conda
https://conda.anaconda.org/conda-forge/noarch/ply-3.11-pyhd8ed1ab_3.conda
https://conda.anaconda.org/conda-forge/osx-arm64/psutil-7.0.0-py312hea69d52_0.conda
https://conda.anaconda.org/conda-forge/noarch/py-cpuinfo-9.0.0-pyhd8ed1ab_1.conda
https://conda.anaconda.org/conda-forge/noarch/pycparser-2.22-pyh29332c3_1.conda
https://conda.anaconda.org/conda-forge/noarch/pyjwt-2.9.0-pyhd8ed1ab_1.conda
https://conda.anaconda.org/conda-forge/noarch/pyparsing-3.2.3-pyhd8ed1ab_1.conda
https://conda.anaconda.org/conda-forge/noarch/pysocks-1.7.1-pyha55dd90_7.conda
https://conda.anaconda.org/conda-forge/noarch/python-tzdata-2025.2-pyhd8ed1ab_0.conda
https://conda.anaconda.org/conda-forge/noarch/pytz-2024.1-pyhd8ed1ab_0.conda
https://conda.anaconda.org/conda-forge/noarch/pyuca-1.2-pyhd8ed1ab_2.conda
https://conda.anaconda.org/conda-forge/osx-arm64/pyyaml-6.0.2-py312h998013c_2.conda
https://conda.anaconda.org/conda-forge/osx-arm64/rpds-py-0.25.1-py312hd3c0895_0.conda
https://conda.anaconda.org/conda-forge/noarch/semver-3.0.4-pyhd8ed1ab_0.conda
https://conda.anaconda.org/conda-forge/noarch/setuptools-80.9.0-pyhff2d567_0.conda
https://conda.anaconda.org/conda-forge/noarch/six-1.17.0-pyhd8ed1ab_0.conda
https://conda.anaconda.org/conda-forge/noarch/sniffio-1.3.1-pyhd8ed1ab_1.conda
https://conda.anaconda.org/conda-forge/noarch/tabulate-0.9.0-pyhd8ed1ab_2.conda
https://conda.anaconda.org/conda-forge/noarch/tomli-2.2.1-pyhd8ed1ab_1.conda
https://conda.anaconda.org/conda-forge/noarch/typing_extensions-4.14.0-pyhe01879c_0.conda
https://conda.anaconda.org/conda-forge/osx-arm64/unicodedata2-16.0.0-py312hea69d52_0.conda
https://conda.anaconda.org/conda-forge/noarch/vine-5.1.0-pyhd8ed1ab_1.conda
https://conda.anaconda.org/conda-forge/noarch/visitor-0.1.3-pyhd8ed1ab_2.conda
https://conda.anaconda.org/conda-forge/noarch/waitress-3.0.2-pyhd8ed1ab_1.conda
https://conda.anaconda.org/conda-forge/noarch/wcwidth-0.2.13-pyhd8ed1ab_1.conda
https://conda.anaconda.org/conda-forge/noarch/wheel-0.45.1-pyhd8ed1ab_1.conda
https://conda.anaconda.org/conda-forge/noarch/zipp-3.22.0-pyhd8ed1ab_0.conda
https://conda.anaconda.org/conda-forge/noarch/amqp-5.2.0-pyhd8ed1ab_2.conda
https://conda.anaconda.org/conda-forge/noarch/bibtexparser-1.4.3-pyhd8ed1ab_0.conda
https://conda.anaconda.org/conda-forge/osx-arm64/cffi-1.17.1-py312h0fad829_0.conda
https://conda.anaconda.org/conda-forge/noarch/click-didyoumean-0.3.1-pyhd8ed1ab_1.conda
https://conda.anaconda.org/conda-forge/noarch/click-plugins-1.1.1-pyhd8ed1ab_1.conda
https://conda.anaconda.org/conda-forge/noarch/clickclick-20.10.2-pyhd8ed1ab_1.conda
https://conda.anaconda.org/conda-forge/noarch/dnspython-2.7.0-pyhff2d567_1.conda
https://conda.anaconda.org/conda-forge/osx-arm64/fonttools-4.58.2-py312h998013c_0.conda
https://conda.anaconda.org/conda-forge/osx-arm64/freetype-2.13.3-hce30654_1.conda
https://conda.anaconda.org/conda-forge/osx-arm64/gmpy2-2.2.1-py312h524cf62_0.conda
https://conda.anaconda.org/conda-forge/noarch/h2-4.2.0-pyhd8ed1ab_0.conda
https://conda.anaconda.org/conda-forge/noarch/importlib-metadata-8.7.0-pyhe01879c_1.conda
https://conda.anaconda.org/conda-forge/noarch/importlib_resources-6.5.2-pyhd8ed1ab_0.conda

```
https://conda.anaconda.org/conda-forge/noarch/jinja2-3.1.6-pyhd8ed1ab_0.conda
https://conda.anaconda.org/conda-forge/osx-arm64/libblas-3.9.0-31_h10e41b3_openblas.conda
https://conda.anaconda.org/conda-forge/noarch/marshmallow-4.0.0-pyhd8ed1ab_0.conda
https://conda.anaconda.org/conda-forge/osx-arm64/openldap-2.6.10-hbe55e7a_0.conda
https://conda.anaconda.org/conda-forge/osx-arm64/pillow-11.2.1-py312h50aef2c_0.conda
https://conda.anaconda.org/conda-forge/noarch/pip-25.1.1-pyh8b19718_0.conda
https://conda.anaconda.org/conda-forge/noarch/prompt-toolkit-3.0.51-pyha770c72_0.conda
https://conda.anaconda.org/conda-forge/osx-arm64/pyobjc-core-11.0-py312hb9d441b_0.conda
https://conda.anaconda.org/conda-forge/noarch/python-dateutil-2.9.0.post0-pyhff2d567_1.conda
https://conda.anaconda.org/conda-forge/noarch/referencing-0.36.2-pyh29332c3_0.conda
https://conda.anaconda.org/conda-forge/osx-arm64/sqlalchemy-1.4.54-py312hea69d52_0.conda
https://conda.anaconda.org/conda-forge/noarch/tqdm-4.67.1-pyhd8ed1ab_1.conda
https://conda.anaconda.org/conda-forge/noarch/typing-extensions-4.14.0-h32cad80_0.conda
https://conda.anaconda.org/conda-forge/noarch/typing-inspection-0.4.1-pyhd8ed1ab_0.conda
https://conda.anaconda.org/conda-forge/noarch/werkzeug-2.2.3-pyhd8ed1ab_0.conda
https://conda.anaconda.org/conda-forge/noarch/wtforms-3.2.1-pyhd8ed1ab_1.conda
https://conda.anaconda.org/conda-forge/noarch/annotated-types-0.7.0-pyhd8ed1ab_1.conda
https://conda.anaconda.org/conda-forge/noarch/email-validator-2.2.0-pyhd8ed1ab_1.conda
https://conda.anaconda.org/conda-forge/noarch/flexcache-0.3-pyhd8ed1ab_1.conda
https://conda.anaconda.org/conda-forge/noarch/flexparser-0.4-pyhd8ed1ab_1.conda
https://conda.anaconda.org/conda-forge/osx-arm64/fontconfig-2.15.0-h1383a14_1.conda
https://conda.anaconda.org/conda-forge/noarch/freetype-py-2.3.0-pyhd8ed1ab_0.tar.bz2
https://conda.anaconda.org/conda-forge/noarch/importlib-resources-6.5.2-pyhd8ed1ab_0.conda
https://conda.anaconda.org/conda-forge/noarch/jsonschema-specifications-2025.4.1-pyh29332c3_0.co
https://conda.anaconda.org/conda-forge/osx-arm64/libcblas-3.9.0-31_hb3479ef_openblas.conda
https://conda.anaconda.org/conda-forge/osx-arm64/liblapack-3.9.0-31_hc9a63f6_openblas.conda
https://conda.anaconda.org/conda-forge/osx-arm64/libpq-17.5-h6896619_0.conda
https://conda.anaconda.org/conda-forge/noarch/mako-1.3.10-pyhd8ed1ab_0.conda
https://conda.anaconda.org/conda-forge/noarch/marshmallow-sqlalchemy-1.4.2-pyhd8ed1ab_0.conda
https://conda.anaconda.org/conda-forge/noarch/pbr-6.1.1-pyhd8ed1ab_1.conda
https://conda.anaconda.org/conda-forge/noarch/prompt_toolkit-3.0.51-hd8ed1ab_0.conda
https://conda.anaconda.org/conda-forge/osx-arm64/pydantic-core-2.33.2-py312hd3c0895_0.conda
https://conda.anaconda.org/conda-forge/osx-arm64/pyobjc-framework-cocoa-11.0-py312hb9d441b_0.con
https://conda.anaconda.org/conda-forge/noarch/reference-handler-0.9.1-pyhd8ed1ab_0.tar.bz2
https://conda.anaconda.org/conda-forge/noarch/swagger-ui-bundle-1.1.0-pyhd8ed1ab_1.conda
https://conda.anaconda.org/conda-forge/noarch/sympy-1.14.0-pyh2585a3b_105.conda
https://conda.anaconda.org/conda-forge/osx-arm64/zstandard-0.23.0-py312hea69d52_2.conda
https://conda.anaconda.org/conda-forge/noarch/alembic-1.16.1-pyhd8ed1ab_0.conda
https://conda.anaconda.org/conda-forge/osx-arm64/cairo-1.18.4-h6a3b0d2_0.conda
https://conda.anaconda.org/conda-forge/noarch/click-repl-0.3.0-pyhd8ed1ab_0.conda
https://conda.anaconda.org/conda-forge/noarch/jsonschema-4.24.0-pyhd8ed1ab_0.conda
https://conda.anaconda.org/conda-forge/osx-arm64/numpy-2.2.6-py312h7c1f314_0.conda
https://conda.anaconda.org/conda-forge/noarch/pint-0.24.4-pyhd8ed1ab_1.conda
https://conda.anaconda.org/conda-forge/noarch/pydantic-2.11.4-pyh3cfb1c2_0.conda
https://conda.anaconda.org/conda-forge/noarch/pyperclip-1.9.0-pyh534df25_3.conda
https://conda.anaconda.org/conda-forge/noarch/stevedore-5.4.1-pyhd8ed1ab_0.conda
https://conda.anaconda.org/conda-forge/noarch/urllib3-2.4.0-pyhd8ed1ab_0.conda
```

```
https://conda.anaconda.org/conda-forge/noarch/botocore-1.38.31-pyge310_1234567_0.conda
https://conda.anaconda.org/conda-forge/osx-arm64/contourpy-1.3.2-py312hb23fbb9_0.conda
https://conda.anaconda.org/conda-forge/osx-arm64/libboost-python-1.86.0-py312h72cd453_3.conda
https://conda.anaconda.org/conda-forge/osx-arm64/librdkit-2025.03.3-hab0a279_0.conda
https://conda.anaconda.org/conda-forge/osx-arm64/openbabel-3.1.1-py312h2eeb2e4_9.conda
https://conda.anaconda.org/conda-forge/osx-arm64/pandas-2.2.3-py312hcd31e36_1.conda
https://conda.anaconda.org/conda-forge/noarch/patsy-1.0.1-pyhd8ed1ab_1.conda
https://conda.anaconda.org/conda-forge/osx-arm64/pycairo-1.28.0-py312h0437987_0.conda
https://conda.anaconda.org/conda-forge/osx-arm64/pycifrw-4.4.6-py312h024a12e_2.conda
https://conda.anaconda.org/conda-forge/noarch/qcelemental-0.29.0-pyhd8ed1ab_1.conda
https://conda.anaconda.org/conda-forge/noarch/requests-2.32.3-pyhd8ed1ab_1.conda
https://conda.anaconda.org/conda-forge/osx-arm64/scipy-1.15.2-py312h99a188d_0.conda
https://conda.anaconda.org/conda-forge/osx-arm64/spglib-2.6.0-py312h3daaf81_0.conda
https://conda.anaconda.org/conda-forge/osx-arm64/matplotlib-base-3.10.3-py312hdbc7e53_0.conda
https://conda.anaconda.org/conda-forge/noarch/pubchempy-1.0.4-pyhd8ed1ab_1.conda
https://conda.anaconda.org/conda-forge/noarch/qcportal-0.54.1-pyhd8ed1ab_0.conda
https://conda.anaconda.org/conda-forge/noarch/requests-toolbelt-1.0.0-pyhd8ed1ab_1.conda
https://conda.anaconda.org/conda-forge/noarch/rlpycairo-0.2.0-pyhd8ed1ab_0.conda
https://conda.anaconda.org/conda-forge/noarch/s3transfer-0.13.0-pyhd8ed1ab_0.conda
https://conda.anaconda.org/conda-forge/noarch/seekpath-2.1.0-pyhd8ed1ab_1.conda
https://conda.anaconda.org/conda-forge/osx-arm64/statsmodels-0.14.4-py312h755e627_0.conda
https://conda.anaconda.org/conda-forge/noarch/boto3-1.38.31-pyhd8ed1ab_0.conda
https://conda.anaconda.org/conda-forge/osx-arm64/reportlab-4.4.1-py312hea69d52_0.conda
https://conda.anaconda.org/conda-forge/noarch/seamm-dashboard-client-2024.6.27-pyhff2d567_0.cond
https://conda.anaconda.org/conda-forge/noarch/seamm-util-2024.8.22-pyhd8ed1ab_1.conda
https://conda.anaconda.org/conda-forge/noarch/kombu-5.5.4-pyha770c72_0.conda
https://conda.anaconda.org/conda-forge/osx-arm64/rdkit-2025.03.3-py312had33d2d_0.conda
https://conda.anaconda.org/conda-forge/noarch/seamm-widgets-2024.10.10-pyhd8ed1ab_0.conda
https://conda.anaconda.org/conda-forge/noarch/celery-5.5.3-pyhe01879c_0.conda
https://conda.anaconda.org/conda-forge/noarch/molsystem-2025.5.19-pyhd8ed1ab_0.conda
https://conda.anaconda.org/conda-forge/noarch/flask-2.2.5-pyhd8ed1ab_0.conda
https://conda.anaconda.org/conda-forge/noarch/seamm-ff-util-2025.5.26-pyhd8ed1ab_0.conda
https://conda.anaconda.org/conda-forge/noarch/connexion-2.14.2-pyhd8ed1ab_1.conda
https://conda.anaconda.org/conda-forge/noarch/flask-bootstrap-3.3.7.1-py_0.tar.bz2
https://conda.anaconda.org/conda-forge/noarch/flask-compress-1.17-pyhd8ed1ab_1.conda
https://conda.anaconda.org/conda-forge/noarch/flask-cors-6.0.0-pyhe01879c_0.conda
https://conda.anaconda.org/conda-forge/noarch/flask-jwt-extended-4.5.3-pyhd8ed1ab_0.conda
https://conda.anaconda.org/conda-forge/noarch/flask-mail-0.10.0-pyhd8ed1ab_0.conda
https://conda.anaconda.org/conda-forge/noarch/flask-marshmallow-1.3.0-pyhd8ed1ab_0.conda
https://conda.anaconda.org/conda-forge/noarch/flask-moment-1.0.6-pyhd8ed1ab_1.conda
https://conda.anaconda.org/conda-forge/noarch/flask-sqlalchemy-3.0.3-pyhd8ed1ab_0.conda
https://conda.anaconda.org/conda-forge/noarch/flask-wtf-1.2.2-pyhd8ed1ab_1.conda
https://conda.anaconda.org/conda-forge/noarch/flask-authorize-0.2.6-pyhd8ed1ab_0.tar.bz2
https://conda.anaconda.org/conda-forge/noarch/seamm-datastore-2024.6.2-pyhd8ed1ab_0.conda
https://conda.anaconda.org/conda-forge/noarch/seamm-2025.5.27-pyhd8ed1ab_0.conda
https://conda.anaconda.org/conda-forge/noarch/seamm-dashboard-2024.6.2-pyhd8ed1ab_0.conda
```

References

(1) Jónsson, H.; Mills, G.; Jacobsen, K. W. *Classical and Quantum Dynamics in Condensed Phase Simulations*; pp 385–404.

(2) Henkelman, G.; Jónsson, H. Improved tangent estimate in the nudged elastic band method for finding minimum energy paths and saddle points. *The Journal of Chemical Physics* **2000**, *113*, 9978–9985.

(3) Makri, S.; Ortner, C.; Kermode, J. R. A preconditioning scheme for minimum energy path finding methods. *The Journal of Chemical Physics* **2019**, *150*, 094109.

(4) Smidstrup, S.; Pedersen, A.; Stokbro, K.; Jónsson, H. Improved initial guess for minimum energy path calculations. *The Journal of Chemical Physics* **2014**, *140*, 214106.

(5) Henkelman, G.; Uberuaga, B. P.; Jónsson, H. A climbing image nudged elastic band method for finding saddle points and minimum energy paths. *The Journal of Chemical Physics* **2000**, *113*, 9901–9904.

(6) Wang, L.-P.; Song, C. Geometry optimization made simple with translation and rotation coordinates. *The Journal of Chemical Physics* **2016**, *144*, 214108.

(7) Larsen, A. H. et al. The atomic simulation environment—a Python library for working with atoms. *Journal of Physics: Condensed Matter* **2017**, *29*, 273002.

(8) Strachan, A.; van Duin, A. C. T.; Chakraborty, D.; Dasgupta, S.; Goddard, W. A. Shock Waves in High-Energy Materials: The Initial Chemical Events in Nitramine RDX. *Phys. Rev. Lett.* **2003**, *91*, 098301.

(9) Singh, S. K.; Srinivasan, S. G.; Neek-Amal, M.; Costamagna, S.; van Duin, A. C. T.; Peeters, F. M. Thermal properties of fluorinated graphene. *Phys. Rev. B* **2013**, *87*, 104114.

(10) Shan, T.-R.; Wixom, R. R.; Thompson, A. P. *Nanoscale Void-Enhanced Initiation in Hexanitrostilbene: Reactive Molecular Dynamics Simulations.*; 2014.

(11) Smith, J. S.; Nebgen, B. T.; Zubatyuk, R.; Lubbers, N.; Devereux, C.; Barros, K.; Tretiak, S.; Isayev, O.; Roitberg, A. E. Approaching coupled cluster accuracy with a general-purpose neural network potential through transfer learning. *Nature Communications* **2019**, *10*, 2903.

(12) Devereux, C.; Smith, J. S.; Huddleston, K. K.; Barros, K.; Zubatyuk, R.; Isayev, O.; Roitberg, A. E. Extending the Applicability of the ANI Deep Learning Molecular Potential to Sulfur and Halogens. *Journal of Chemical Theory and Computation* **2020**, *16*, 4192–4202, PMID: 32543858.

(13) Fink, T.; Reymond, J.-L. Virtual Exploration of the Chemical Universe up to 11 Atoms of C, N, O, F: Assembly of 26.4 Million Structures (110.9 Million Stereoisomers) and Analysis for New Ring Systems, Stereochemistry, Physicochemical Properties, Compound Classes, and Drug Discovery. *Journal of Chemical Information and Modeling* **2007**, *47*, 342 – 353.

(14) Nguyen, T. L.; Thorpe, J. H.; Bross, D. H.; Ruscic, B.; Stanton, J. F. Unimolecular Reaction of Methyl Isocyanide to Acetonitrile: A High-Level Theoretical Study. *The Journal of Physical Chemistry Letters* **2018**, *9*, 2532–2538, PMID: 29697985.

(15) Smith, J. S.; Nebgen, B.; Lubbers, N.; Isayev, O.; Roitberg, A. E. Less is more: Sampling chemical space with active learning. *The Journal of Chemical Physics* **2018**, *148*, 241733.

(16) Smith, J. S.; Nebgen, B. T.; Zubatyuk, R.; Lubbers, N.; Devereux, C.; Barros, K.; Tretiak, S.; Isayev, O.; Roitberg, A. E. Approaching coupled cluster accuracy with a general-purpose neural network potential through transfer learning. *Nature Communications* **2019**, *10*, 2903.

(17) Devereux, C.; Smith, J. S.; Huddleston, K. K.; Barros, K.; Zubatyuk, R.; Isayev, O.; Roitberg, A. E. Extending the Applicability of the ANI Deep Learning Molecular Potential to Sulfur and Halogens. *Journal of Chemical Theory and Computation* **2020**, *16*, 4192–4202, PMID: 32543858.
